# Programming Mat-Poka-yoke system control effect of unsteady convection-diffusion behavior for plastic manufacturing mathematically

**DOI:** 10.1371/journal.pone.0336617

**Published:** 2026-04-08

**Authors:** Ahmed M. Abed, Ali AlArjani, Laila F. Seddek, Mohamed Salah, Ola Ragb, Rania B. M. Amer

**Affiliations:** 1 Department of Industrial Engineering, College of Engineering, Prince Sattam bin Abdulaziz University, Al-Kharj, Saudi Arabia; 2 Department of Mathematics, College of Science and Humanities in Al-Kharj, Prince Sattam Bin Abdul-Aziz University, Al-Kharj, Saudi Arabia; 3 Department of Physics and Engineering Mathematics, Faculty of Engineering, Zagazig University, Egypt; University of Sharjah, UNITED ARAB EMIRATES

## Abstract

The blowing and drying stages in the plastic manufacturing process generate many defects due to a lack of accuracy in estimating thermal convection and its diffusion control. Therefore, dual digital poka-yoke simulator phases have been built to describe the unsteady state of convection-diffusion mathematically (i.e., parabolic behavior), and converted to ordinary type using a discrete singular convolution transformer. The numerical solution improves the mechanism of JRNS (Jidoka recruit’s network system) to be easily programmed to be highly controlled. The mathematical poka-yoke simulator formulation relies on Runge-Kutta 4^th^ order and five-stage fourth-order robust stability protective Runge-Kutta (SSP-RK54) schemes as discussed in 1^st^ phase and called Mat-Poka-Yoke system (Mat-PYS) alternating between three stages. The accuracy of the Mat-PYS has been tested via measuring the rate of convergence, the absolute error, the L2 error, and the L∞ error, with errors up to $\text{1}\times {\text{1}\text{0}}^{-\text{5}}$. The obtained outcomes are described both in tabular and graphical form, which almost includes the validity of these mechanisms to hold on to the precision, efficiency, simplicity, and applicability for solving convection-diffusion equations. The applicability emphasized via parametric analysis to debate the effect of convective velocities, diffusion coefficients, and time at different locations on results to resist the defect causes generation as demonstrated in 2^nd^ phase. The OEE for plastic injection machine process has been improved from 76.6% to 88.9% when controlled by proposed mechanism, and products quality improved to 5.2 sigma level.

## 1. Introduction

Usually, conventional approximation relationships involving dimensionless numbers are used to find convective and diffusion transfer coefficients. These approximations were created in the past to circumvent the challenges involved in computing massive series expansions and the expansion coefficients that go along with them, which are provided by transcendental equations. These challenges have been eliminated, though, with the advancement of better computer methods, making such computations now simple. The use of approximate relationships is avoided in favor of an iterative process that makes use of current computer capabilities as cited by Abed et al., (2022) [1]. It is important to note that, as Zhao et al. (2014) [2] point out, foods are typically dried for industrial purposes to reduce moisture levels to a point where deteriorative enzymatic processes and microbial spoiling are decreased. Accurate diffusion and convective transfer coefficient calculations are necessary to enable precise designs when building accurate digital twins that lead to high control systems. The blowing and drying stage after plastic injection process into the mold is an important and time-consuming stage that affects productivity and rushing it may produce defective products. The problem lies in the lack of programming the injection machine with accurate equations for calculating the heat transfer and the accompanying diffusion during the drying process as discussed by AMAbed et al., (2023) [3].

By viewing drying as a process that combines a vapor and condensed situation, the interactive modelling technique (i.e., digital twins) has been used to determine the convective transfer coefficient as discussed by Compaore et al., (2017) accurately [4]. Also, fitting the drying operation form to be a logarithmic built over a straight line is the most used technique. So, the first assumption in the sequential solutions is that the slope of the curve can be used to find the diffusion coefficient for simple one-dimensional shapes like slices or cylinders. Many scientific disciplines, including industrial engineering, applied mathematics, chemistry, biology, fluid motion, physics engineering, hydraulics, and many more, use the convection-diffusion equation, a semi-linear parabolic partial differential equation, to explain complex phenomena [5,6]. Convection-diffusion equation modelling gives rise to a wide range of issues. In order to achieve greater accuracy levels, researchers attempt to solve these equations numerically and/or analytically using a variety of methods and formulas. Among these, Yadav and Kumar [7] found a solution to the one-dimensional convective diffusion problem by using the homotopy perturbation technique (HPM). It was done by Demetriou et al. [8] to fully classify the Lie groups of two- and three-dimensional classes of nonlinear diffusion-convection equations. By using the homotopy perturbation technique (HPM), Kumar and Singh [9] were able to solve Kolmogorov-Petrovsly-Piskunov equations. Kim [10] presented a general 1D analytic solution of the convection-diffusion-reaction-source equation by applying a one-sided Laplace transform. Gupta et al. [11] applied a variety of linear and nonlinear convection-diffusion problems can be solved using the Laplace transform and/or homotopy perturbation method. Discovering optimal solutions of convection-diffusion equations is not a simple task. So, researchers are attempting to improve an accurate and efficient numerical technique for solving these equations such as finite element (FE) scheme [12, 13], finite difference method [14–16], Lattice Boltzmann technique [17], spectral methods [18,19], finite volume methods [20,21], shifting the least-squares collocation algorithm [22], Galerkin and Decomposition methods [23,24] are a lot applied in numerical analysis. Further, Differential-quadrature method (DQM) [25–32] is a powerful discretization technique for the numerical solution of convection-diffusion patterns. Bellman et al. [25] and Shu [26,27] presented the optimal essentials and developments of DQM as well as its main applications in engineering. Facility of application, accuracy, efficiency, and low-cost computational make this technique favored. But the accuracy results of DQM based on choice the grid points’ number for the tested function. Bellman et al. [25] introduced different of bases functions whether spline or Sinc, and Lagrange polynomials for interpolation, modified cubic B-splines, and radial base. These shape functions are used for solving such problems [26–32]. Also, Ragb et al. [33–37] offered DQ mechanisms relies on several base functions like PDQM that focuses on interpolation of Lagrange, which evolved into two aspects Delta Lagrange (DSCDQM-DLK) and Regularized Shannon kernels(DSCDQM-RSK) mimicked to Cardinal sine (SDQM), and proved how the various test functions control the efficiency, stability via their convergence of the outcomes. Wang, X. et al., (2020) present sufficient review emphasizes the importance of previous citation [38]. Ji, Y., & Xing, Y. (2023) [39] discuss the unconditional stability case that synchronizes with Mustafa, A. et al., (2023) works [40,41] who present the generalised nonlinear quadrature for novel fractional-order chaotic systems and focus on describing the diffusion behaviour via DQM, which may be an extension to Fedotov, A. (2014) [42] work that presents singular integro-differential equations simplifying the future analysis and tracking the convection-diffusion case. DSCDQM-RSK is a computationally efficient method that requires less CPU time which is essential in industrial application whether solving initial and boundary value problems as emphasized by Ragb et al. (2021) and Abed, A.M. et al., (2024) [43]. However, there is a gap in mathematical and industrial applications fields due to a lack of using discrete singular convolution (DSC) in studying convection-diffusion pattern equations despite its powerful. The numerical approaches employed ODEs system to tackle the parabolic convection-diffusion problem in time and then utilize the various time integration algorithms to solve the resulting system [44–48]. Islam et al. [49] investigated numerical solutions of IVP via FE technique via Series of Taylor procedures. They offered an integration method to approximate the numerical solution of an IVP of DEs. Lima et al. [50] applied Gauss-Legendre quadrature rules with Taylor’s series to get high accurateness when formulate the FEM with Error Correlation to treat the time in Convection-Diffusion-Reaction Equations. Through the use of polynomial DQM, Runge–Kutta 4^th^ order (RK4), and block marching, which investigated non-linearity PDEs by Salah et al., (2013). They noted that the RK4 has efficient computational ODEs in 4^th^ order and gives better results than the current mechanisms if compared with their outcomes accuracy with minimum error and function approximations trials. Therefore, the digital simulator Mat PYS relies on these trials to check validity. Recently, SSP-RK54 method [51–53] is preferred because it diminishes storage size, which leads to fewer accumulation errors. Gottlieb et al., (1991) discuss the conditions for spectral approximations to hyperbolic initial-boundary value problems to present the strong of using the Runge-Kutta and multi-step time discretisations that have strong stability preserving in 2005 [54–56]. The mathematical model used by Abed et al., (2020) [57] based in analysis of Gottlieb et al., (2006) and Salah et al., (2013).

This paper demonstrates DSC relies on three different sets of base functions called as aforementioned above DLK, RSK and RDK [58–61]. to obtain optimal numerical results of (2 + 1)-dimensional for the CDE via converting to the system of ODEs in time. For time discretization, ODEs system is treatment by (RK4) and SSP-RK54 schemes. The precision and validity of the mechanisms is confirmed by comparing the obtained approximate solutions by any of the schemes with the optimal solutions via designing MATLAB code for solving these problems. Also, all the three mechanisms are found converge and stable by calculation of different arithmetical sensitive analysis like convergence percentage and its rate, RMS, MAE, L_2_ and L_∞_ errors [62,65] as agreed upon by many researchers. As a result, the calculated results show how easy it is to implement the suggested method and how accurate, dependable, and efficient it is. To further demonstrate how convective velocities, diffusion coefficients, time, and various locations affect the outcomes, a parametric study is presented and facilities building simulation model like Digital twin describe the behavior of suggested model. To create the digital Mat PYS simulator, we employed two distinct simulation mechanisms. We were able to determine the efficient diffusivity, which ranged from 1.83 × 10^−11^ to 1.18 × 10^−10^ m^2^ s − 1, by using an isothermal model according to the theory of diffusion. The second was an equation model that illustrated the connection between mass and heat exchanges. Both mechanisms are based on solving DSC algorithms. The energy required to activate it is 49.6 kJ mol^−1^. The coefficient of thermal transfer ranged from 46.38 to 214.79 W $m^{-\;\text{2}\;}$◦C^− 1^, and the mass transfer coefficient ranged from 4.038 × 10^− 5^ to 2.137 × 10^− 4^ kg $s^{-\;\text{1}\;}$m^− 2^. The convection-diffusion equation was solved in multidimension to see which of the suggested mathematical models best described the product’s drying kinetics. Using a series of computer simulations, A. Baykasoglu and S. Akpinar (2015) [66] demonstrate the resilience, efficacy, and performance of the WSA algorithm by focusing on the degree of convergence reached and its ability to manage problems with premature convergence and entrapment in local optima. Saka et al. (2016) [67] emphasise that metaheuristic methods are strong and dependable tools for solving design optimisation problems in a variety of fields, particularly prediction missions. They also have an effective relationship to present optimal solutions if fed with numerical values. A broad strategy that can be combined with a variety of subset assessment methods is the harmony search (HS). The overall complexity of the search process is decreased by taking advantage of HS’s minimalism. Because HS is uncertain, HS can detect numerous solutions and avoid the local one. Along with detailed analyses of the proposed enhancements, the final method is contrasted with those that depend on HC, genetic algorithms, and particle swarm optimisation as discussed by Kennedy, J. et al. (1995) [68], Zong Woo Geem (2016) [69]. The proposed mechanisms are compared with the Jidoka Recruits Network System (JRNS), which integrates two meta-heuristic methods: Harmony Search (HS) and Weighted Superposition Attraction (WSPA). This discussion focuses on how mathematical results can be used to adjust operating parameters to manage unsteady state behaviour, such as convection-diffusion, to keep plastic products within specifications and reduce defects [70].

There are some interesting papers that focus on studying the diffusion and convection in nanofluid based on metal particles, while our work uses molten material sensitive to heat like plastic. Nisha, S.S. et al. (2025) and De, Poulomi (2025) emphasise the integrity of the mathematical approach when using the 5th-order Runge–Kutta–Fehlberg method to transform partial differential equations (PDEs) into nonlinear ordinary differential equations (ODEs) relies on shooting technique [71,72]. Rajeswari, P.M. et al. (2024) examine the bioconvection phenomenon for enhancing thermal transmission rate [73], but what if they integrate the poka-yoke system in their control system? It will be great if researchers compare our results with this methodology to show the accuracy according to the convergence index, where this study based on manufacturing distinguishes for industrial purposes demanding substantial heat transfer capabilities, while in our product the heat must be under control because its increasing or decreasing will lead to product defect.

The paper discusses the problem of lacking control in the injection process through the blowing and drying stages, which leads to the proposed control system amalgam between accuracy and rapid decision to adjust the injection process parameters to rescue products to be defective. Therefore, the proposed mechanism gathers accuracy via adopting a mathematical solution for unsteady behaviour of convection-diffusion phenomena and meets rapidity via using a metaheuristic system called JRNS (Jidoka recruit’s network system), which consists of hybridising two heuristic algorithms HS (harmonic search) and WSPA (weighted superposition attraction algorithm). The research gap discussed in **section (2)**, which highlights the limited or unexplored employed discrete singular convolution (DSC) to simplify this parabolic behaviour. Therefore, building a digital Mat-PY simulator from two sequential phases to convert the parabolic unsteady phenomena to ordinary type to feed its solution to the 2^nd^ phase that predicts the deviation in metaheuristic parameters that set the injection process as discussed in **section (3)**. The Innovative descriptive Convection-diffusion behavior Results discussed in **section (4)** and emphasizing the defect causes elimination discussed in **section (5)**. The conclusion demonstrates the significant results and validity of proposed mechanism of controlling injection machines has been discussed in **section (6)**. Finally, The future work declares the limitation of the study on injection process, but the mathematical modeling that relies on transferring the OPDs to ODEs is wide used in many sectors as discussed in Ref. [71–73] and advocate the researchers to compare their work with the current JRNS as a future work discussed in **section (7)**.

## 2. The research gap

Improving the control system of machining requires an accurate numerical solution for highly complex behaviour represented by parabolic equations. The first comprehensive analysis of matheuristic modelling strikes a balance between mathematical accuracy and the decision-making speed of heuristic techniques for defect resistance during the blowing and drying process, which leaves four waste’ types that lead to process malfunction. However, there is a notable gap in integrating mathematical and industrial applications and simplifying unsteady behaviour via discrete singular convolution (DSC) in the study of convention-diffusion pattern equations. The proposed mechanism is called the Mat-Poka-Yoke simulator (Mat-PYS), which is distinguished by rapid computing. using DSC-DQM-(RSK or RDK) algorithms due to limited or unexplored efficiency in controlling convection-diffusion phenomena. The DSC algorithm has proven useful in Fokker-Planck and sine-Gordon equations. Therefore, candidates integrate with JRNS and measure the OEE of manufacturing process. This research focuses on the rapid adaptiveness of a drying system with convection-diffusion distribution, distinguished by rapid computing.

## 3. Phase I: the digital Mat-PY simulator formulation

The simulator consists of two phases; the 1^st^ is interested in getting functions to describe the behaviour of thermal convection-diffusion at specific domains and fractional parameters that present solutions compared with exact to test accuracy. The 2^nd^ phase consists of two sequential stages; the 1^st^ focuses on cooling the mould surface, and the 2^nd^ focuses on sucking hot air from products via the drying process for the thin layer.

**Fig 1:**
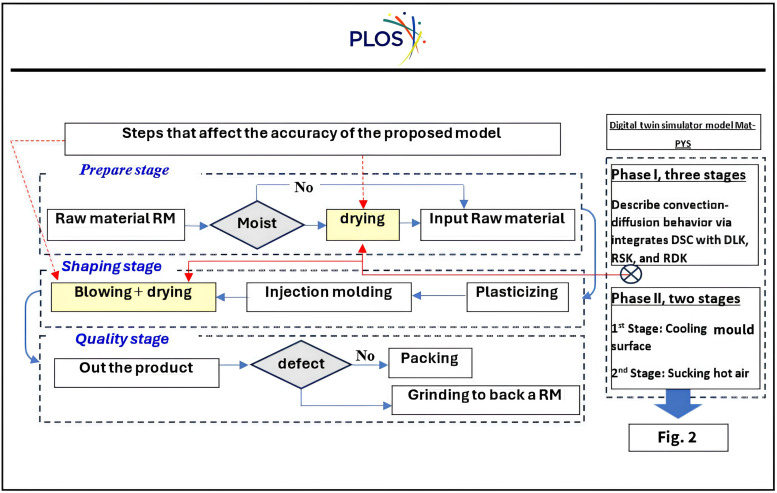
Plastic injection manufacturing.

Thin-layer of plastic products drying research at lab scale and process modelling are basically necessary for dryer design and optimisation. As explained by Myllymaa et al. (2019) [74], this allows one to estimate the duration of drying without defective products generated for the dryer’s area or volume computation and, as a result, receive outcomes like yield and processing capacity as quoted in Silva et al. (2023) [75]. Furthermore, mathematical models are equally significant for forecasting the energy consumption of the drying process of thin plastic products, such as pipes, to balance the necessary quality of the dried products during blowing with operating costs, as discussed by Jimoh et al., (2023) [76] and Abed, A. M. (2024) [77]. Fig 1 illustrates the Plastic injection stages and highlights the Steps that affect the accuracy of the proposed model.

For the purpose of validating Mat-PYS, a (2 + 1) dimensional convection-diffusion equation with general boundary and primary conditions is investigated for the first time by Liu et al. [78] and emphasized by Xuan, H. et al. (2025) [79]. The digital simulator relies on generated solutions by three distinct sets of base functions. The governing equations for the description of unsteady state of convection-diffusion equation in (2 + 1) dimensions can be expressed as:


$$\frac{\partial u}{\partial t}-\alpha_{x}\frac{\partial^{\text{2}}u}{\alpha x^{\text{2}}}-\alpha_{y}\frac{\partial^{\text{2}}u}{\alpha y^{\text{2}}}+\beta_{x}\frac{\partial u}{\partial x}+\beta_{Y}\frac{\partial u}{\partial y}=\text{0}\;in\;\Gamma \times (\text{0},\;T]$$ (1)

where Γ is computational domain $\Gamma =\left[x_{\text{1}},\;x_{\text{2}}\right]\times \left[y_{\text{1}},\;y_{\text{2}}\right]\;\subset R^{\text{2}}$, is time interval. u(x, y, t) is heat or vorticity. αx > 0, αy > 0 are diffusion coefficients. $\beta_{x}$ and $\beta_{Y}$ are constant convective velocities.

Boundary conditions are expressed as:


$$C_{\text{1}}u+D_{\text{1}}\frac{\partial u}{\partial x}=W_{\text{1}}(y,t)\ldots \;\forall (x_{\text{1}},\;y,\;t)$$ (2)

$$C_{\text{2}}u+D_{\text{2}}\frac{\partial u}{\partial x}=W_{\text{2}}(y,t)\ldots \;\forall (x_{\text{2}},\;y,\;t)$$ (3)

$$C_{\text{3}}u+D_{\text{3}}\frac{\partial u}{\partial y}=W_{\text{3}}(x,t)\ldots \;\forall (x,\;y_{\text{1}},\;t),$$ (4)

$$C_{\text{4}}u+D_{\text{4}}\frac{\partial u}{\partial y}=W_{\text{4}}(x,t)\ldots \;\forall (x,\;y_{\text{2}},\;t),$$ (5)

Initial condition is introduced as:


u(x,y,0)= Ψ(x,y), (6)

Where $C_{i},\;D_{i},\;W_{i},\;\forall \;i=\text{1},\text{2},\text{3},\text{4}\;$ and Ψ(x, y) are known functions.

The presented boundary conditions are idealized and assumed:

- Linearity (i.e., linear combinations of u and its derivatives).
- Time and space dependence only in the source terms $W_{i}$ not in the coefficients $C_{i},\;D_{i},\;\forall \;i=\text{1},\text{2},\text{3},\text{4}$.
- Constant convective or diffusive coefficients.

**Fig 2:**
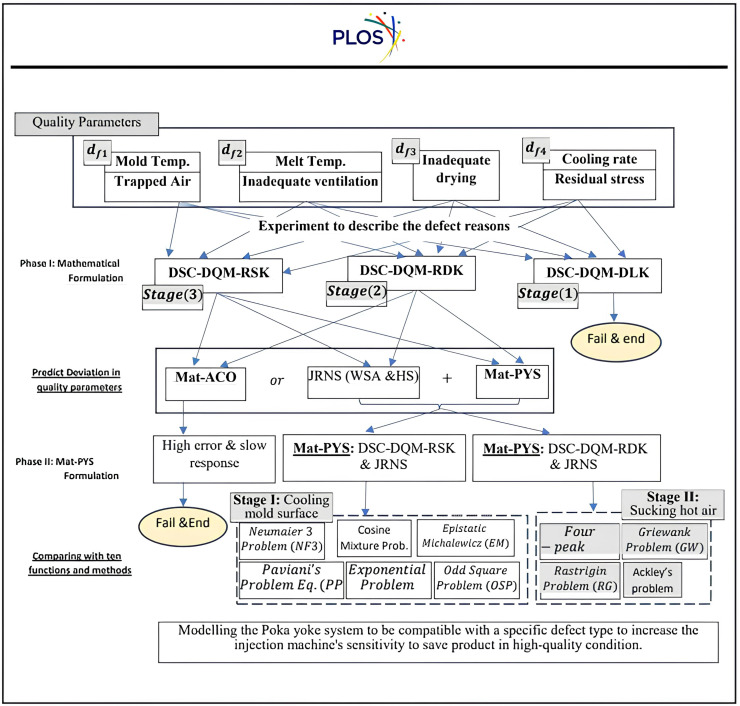
The schema of framework for the manuscript.

Four wastes lead to defects in the manufacturing process caused by a lack of setting the behaviour of heat convection-diffusion phenomena in drying plastic during the injection process. The research focuses on tracking the flow lines, black spots, delamination, and warpage defect types. The main reason that leads to flow line ($d_{f1}$) defects on the bottle’s surface is uneven cooling of molten plastic, which is injected in different mould thicknesses or at different rates. The 2^nd^ defect is Black spots and burn marks ($d_{f2}$), which are caused by trapped air while injection moulding, heat-induced material breakdown, excessive melt temperatures, and inadequate ventilation. The 3^rd^ defect is delamination ($d_{f3}$), which appears between the bottle body and the neck of the lid and is primarily caused by inadequate drying of raw materials. The last defect is Warpage ($d_{f4}$) in plastic parts caused by uneven shrinkage due to varying cooling rates and residual stress, triggered by mould temperature variations and incorrect processing parameters. The Mat-PYS, divided into two phases, includes accuracy via forming a mathematical model and interest in solving high-stable and accurate numerical solutions to express the convection-diffusion equation via Runge-Kutta 4th order and SSP-RK54 methods that discover their efficiency in diagnosing the defects, as demonstrated in Fig 2, and use the metaheuristic methods to accelerate the setting of manufacturing parameters to maintain product quality in a high-quality manufacturing environment. The quick response to set these parameters via Mat-PYS compared with JRNS [70,93] and Mat-ACO [95].

### 3.1. Methods used in building digital Mat-PYS solutions

In this section, DQM has been briefly reviewed then Mat-PYS based on three different sets of shape functions (DLK, RSK and RDK) is derived depending on DQM. Also, RK4 and SSP-RK54 schemes are discussed. All these mechanisms are used to solve convection-diffusion equations. Let the domain a ≤ x ≤ b of a problem be approximated using M grids; x_1_ = a, x_2_,…,x_M_ = b. When the DQM is applied, a weighted linear sum of the function values at all discrete nodes in the domain can be used to approximate the various order derivatives of a function at a designated point (i.e., specific node). For example, the 1^st^ and 2^nd^ derivative of f (x) at specific point $x_{i}$ can be discretised via DQM as expressed in Eqn. (7) [27]:


$$\begin{array}{c} f_{x}\;\left(x_{i},\;y_{j},t\right)=\sum_{k=\text{1}}^{M}\overset{x}{\underset{ik}{P}}f\left(x_{k},\;y_{j},t\right),\;\&\;f_{xx}\;\left(x_{i},\;y_{j},t\right)=\sum_{k=\text{1}}^{Mx}\overset{xx}{\underset{ik}{P}}f\left(x_{k},\;y_{j},t\right), \end{array}$$ (7)

Where $\overset{x}{\underset{ik}{P}}$ and $\overset{xx}{\underset{ik}{P}}$ are the 1^st^ and 2^nd^ weighting coefficients [23].

#### 3.1.1. Stage I: Discrete-Singular-Convolution Differential Quadrature Method (DSCDQM).

In the context of distribution theory, a singular convolution can be defined by [58–65,74–76]:


$$\text{E}(t)=(\varphi *\eta )(t)=\int_{-\infty }^{\infty }\varphi (t-x)\eta (x)dx,$$ (8)

where φ(t−x) is a unique kernel, while DSC technique defined by utilizing several kernel types, which are used as base functions such that the unidentified f and its derivatives are estimated as weight for linear sum of $f_{i}(i=-M:M)$ over a narrow bandwidth ($X-X_{S},\;X+X_{S})$. Therefore, the choice of type kernels and discretizing the spatial area by uniform nodal points determine efficiency and accuracy results. Three DSC kernels for the convection-diffusion equation are used in this problem as follows:

(a) ***First shape function is Delta Lagrange Kernel (DLK) which defined as***:

$$f\left(X_{i}\right)=\sum_{k=-S}^{S}\frac{\prod_{j=-S}^{S}\left(X_{i}-X_{j}\right)}{\left(X_{i}-X_{k}\right)\prod_{j=-S,\;k\neq j}^{S}\;\left(X_{k}-X_{j}\right)}f\left(X_{k}\right),\;\forall \;(i=-M,\;M),\;M\geq \text{1},$$ (9)

The weighting coefficients $\overset{x}{\underset{ik}{P}}$ and $\overset{xx}{\underset{ik}{P}}$ are given by DSCDQM-DLK which are defined as follows:


$$\begin{array}{c} \overset{x}{\underset{ik}{P}}\left\{ \begin{array}{cc} \frac{\text{1}}{\left(X_{i}-X_{k}\right)}\prod_{j=-S,\;k\neq i,\;k}^{S}\frac{\left(X_{i}-X_{j}\right)}{\left(X_{k}-X_{j}\right)} & i\neq k\\ -\sum_{k=-S,\;k\neq i}^{-S}\overset{x}{\underset{ik}{P}} & i=k \end{array}\right.\;,\;\overset{xx}{\underset{ik}{\;P}}\left\{ \begin{array}{cc} \text{2}(\overset{x}{\underset{ik}{P}}.\overset{x}{\underset{ii}{P}}-\frac{\overset{x}{\underset{ik}{P}}}{\left(X_{i}-X_{k}\right)}) & i\neq k\\ -\sum_{k=-S,\;k\neq i}^{-S}\overset{xx}{\underset{ik}{P}} & i=k \end{array}\right.\;, \end{array}$$ (10)

(b) ***Second shape function is Regularized Shannon kernel (RSK) which presented as follows:***

$$f\left(X_{i}\right)=\sum_{k=-S}^{S}\left\langle \frac{\text{sin}\left[\pi (X_{i}-X_{k})/g_{X}\right]}{\left[\pi (X_{i}-X_{k})/g_{X}\right]}e^{\left(\frac{{\left(X_{i}-X_{k}\right)}^{\text{2}}}{\text{2}\gamma^{\text{2}}}\right)}\right\rangle \;f(X_{k}),\;(i=-M,\;M),\;\gamma =\left(d*g_{X}\right)>\text{0},$$ (11)

The weighting coefficients $\overset{x}{\underset{ik}{P}}$ and $\overset{xx}{\underset{ik}{P}}$ for DSCDQM-RSK are written as [58–65,74–76]:


$$\begin{array}{c} \overset{x}{\underset{ik}{P}}=\left\{ \begin{array}{cc} \frac{{\left(-\text{1}\right)}^{i-k}}{g_{X}(i-k)}e^{-\overset{\text{2}}{\underset{X}{g}}(\frac{{\left(i-k\right)}^{\text{2}}}{\text{2}\gamma^{\text{2}}})} & i\neq k\\ \;\;\;\;\;\;\;\;\;\;\;\;\;\;\;\;\;\;\;\;\;\;\;\;\;\;\;\;\;\;\;\;\;\;\;\;\;\;\text{0} & i=k \end{array}\right.\;,\overset{xx}{\underset{ik}{P}}=\left\{ \begin{array}{cc} (\frac{\text{2}{\left(-\text{1}\right)}^{i-k+\text{1}}}{\overset{\text{2}}{\underset{X}{g}}{\left(i-k\right)}^{\text{2}}}+\frac{\text{1}}{\gamma^{\text{2}}})e^{-\overset{\text{2}}{\underset{X}{g}}(\frac{{\left(i-k\right)}^{\text{2}}}{\text{2}\beta^{\text{2}}})} & i\neq k\\ \;\;\;\;\;\;\;\;\;\;\;\;\;\;\;\;\;\;\;\;\;\;\;\;\;\;\;\;\;\;\;\;\;\;\;\;\;\;-\frac{\text{1}}{\gamma^{\text{2}}}-\frac{\pi^{\text{2}}}{\text{3}\overset{\text{2}}{\underset{X}{g}}} & i=k \end{array}\right.\;, \end{array}$$ (12)

Where 2S+1 has a more effective computed bandwidth. Also, γ and d are parameters for computation and regularization, while $g_{x}$ represents a step size.

(c) ***The third shape function, Regularised Dirichlet kernel (RDK), is introduced as follows:***

$$f\left(X_{i}\right)=\sum_{k=-S}^{S}\left\langle \frac{\text{sin}\left[\pi (X_{i}-X_{k})/g_{X}\right]}{\left(\text{2}T+\text{1})\text{tan}[\pi (X_{i}-X_{k})/g_{X}(\text{2}T+\text{1})\right]}e^{\left(\frac{{\left(X_{i}-X_{k}\right)}^{\text{2}}}{\text{2}\gamma^{\text{2}}}\right)}\right\rangle \;f(X_{k}),\;(i=-M,\;M),\;\gamma =\left(d*g_{X}\right)>\text{0},$$ (13)

The weighting coefficients $\overset{x}{\underset{ik}{P}}$ and $\overset{xx}{\underset{ik}{P}}$ for DSCDQM-RDK are expressed as [53–62]:


$$\begin{array}{c} \overset{x}{\underset{ik}{P}}=\left\{ \begin{array}{cc} \frac{\pi {\left(-\text{1}\right)}^{i-k}}{(\text{2}T+\text{1})g_{X}\text{tan}\left[\pi (i-k)/(\text{2}T+\text{1})\right]}e^{-\overset{\text{2}}{\underset{X}{g}}(\frac{{\left(i-k\right)}^{\text{2}}}{\text{2}\gamma^{\text{2}}})}, & i\neq k\\ \text{0} & i=k \end{array},\right.\; \end{array}$$ (14)

$$\begin{array}{c} \overset{xx}{\underset{ik}{P}}=\left\{ \begin{array}{c} {\left(\frac{\text{2}\pi^{\text{2}}{\left(-\text{1}\right)}^{i-k+\text{1}}}{\overset{\text{2}}{\underset{X}{g}}{\left(\text{2}T+\text{1}\right)}^{\text{2}}{sin}^{\text{2}}[\pi (i-k)/(\text{2}T+\text{1})]}+\frac{\text{2}\pi (i-k){\left(-\text{1}\right)}^{i-k+\text{1}}}{{\left(\text{2}T+\text{1})(\gamma \right)}^{\text{2}}tan[\pi (i-k)/(\text{2}T+\text{1})]}\right)e}^{\overset{\text{2}}{\underset{X}{-g}}(\frac{{\left(i-k\right)}^{\text{2}}}{\text{2}\gamma^{\text{2}}})}\\ \;\;\;\;\;\;\;\;\;\;\;\;\;\;\;\;\;\;\;\;\;\;\;\;\;\;\;\;\;\;\;\;\;\;\;\;\;\;\;\;\;\;\;\;\;\;\;\;\;\;\;\;\;\;\;\;\;\;\;\;\;\;\;\;\;\;\;\;\;\;\;\;\;\;\;\;\;\;\;\;\;\;\;\;\;\;\;\;\;\;\;\;\;\;\;\;\;\;\;\;\;\;\;\;\;\;\;\;\;\;\;\;\;\;\;\;\;\;\;\;\;\;\;-\frac{\text{1}}{\gamma^{\text{2}}}-\frac{\pi^{\text{2}}}{\text{3}\overset{\text{2}}{\underset{X}{g}}} \end{array}\right.\;\begin{array}{cc} & i\neq k\\ & i=k \end{array} \end{array}$$ (15)

T is a parameter if **T→∞** RDK transforms into RSK. To implement these methods on (2 + 1) convection-diffusion equations after computing weighting coefficients $\overset{x}{\underset{ik}{P}}$, $\overset{y}{\underset{ik}{P}}$, $\overset{xx}{\underset{ik}{P}}$and $\overset{yy}{\underset{ik}{P}}$. The governing equation is transformed into a system of ODEs in time by substituting Eqn. (7) into (1) as follows:


$${\;\frac{du}{dt}|}_{\left(x_{i}\;,y_{j}\;,\;t\right)}=\alpha_{x}\sum_{k=\text{1}}^{M_{x}}\overset{xx}{\underset{ik}{P}}\;u(x_{k},\;y_{j}\;,\;t)+\alpha_{y}\sum_{k=\text{1}}^{M_{y}}\overset{yy}{\underset{ik}{P}}\;u(x_{i},\;y_{l}\;,\;t)-\beta_{x}\sum_{k=\text{1}}^{M_{x}}\overset{x}{\underset{ik}{P}}\;u\left(x_{k},\;y_{j}\;,\;t\right)-\;\beta_{y}\sum_{k=\text{1}}^{M_{y}}\overset{y}{\underset{ik}{P}}\;u(x_{i},\;y_{l}\;,\;t)$$ (16)

Also, by substitution Eqn. (11) into Eqns. (2–5) the boundary conditions can be written as follows:


$$C_{\text{1}}\;u\left(x_{\text{1}},\;y_{j},\;t\right)+D_{\text{1}}\sum_{k=\text{1}}^{M_{x}}\overset{x}{\underset{\text{1}k}{P}}\;u\left(x_{\text{1}},\;y_{j},\;t\right)=W_{\text{1}}\left(y_{j},\;t\right),$$ (17)

$$C_{\text{2}}\;u\left(x_{\text{2}},\;y_{j},\;t\right)+D_{\text{2}}\sum_{k=\text{1}}^{M_{x}}\overset{x}{\underset{\text{2}k}{P}}\;u\left(x_{\text{2}},\;y_{j},\;t\right)=W_{\text{2}}\left(y_{j},\;t\right),$$ (18)

$$C_{\text{3}}\;u\left(x_{i},\;y_{\text{1}},\;t\right)+D_{\text{3}}\sum_{l=\text{1}}^{M_{y}}\overset{y}{\underset{\text{1}l}{P}}\;u\left(x_{i},\;y_{\text{1}},\;t\right)=W_{\text{3}}\left(x_{i},\;t\right),$$ (19)

$$C_{\text{4}}\;u\left(x_{i},\;y_{\text{2}},\;t\right)+D_{\text{4}}\sum_{l=\text{1}}^{M_{y}}\overset{y}{\underset{\text{2}l}{P}}\;u\left(x_{i},\;y_{\text{2}},\;t\right)=W_{\text{4}}\left(x_{i},\;t\right),$$ (20)

Additionally, the initial and boundary conditions handled by adding it in the governing equations. The system of ODEs solved using the Runge-Kutta 4^th^ order and SSP-RK54 methods to overcome time discretisation during simulation running is as discussed [50–56]:

#### 3.1.2. Stage II: Runge-Kutta 4^th^ order method.

Runge-Kutta 4^th^ order is an arithmetical procedure applied to explain the initial value system [51–56]. This method will give us higher accuracy without making more computation. The formula for RK4 method is explained as:


$$u\left(x_{i},\;y_{j},\;t_{k+\text{1}}\right)=u\left(x_{i},\;y_{j},\;t_{k}\right)+\frac{\text{1}}{\text{6}}\left[\overset{ij}{\underset{\text{1}}{R}}+\text{2}\left(\overset{ij}{\underset{\text{2}}{R}}+\overset{ij}{\underset{\text{3}}{R}}\right)+\overset{ij}{\underset{\text{4}}{R}}\right],$$ (21)

$$\overset{ij}{\underset{\text{1}}{\text{Where }R}}=\Delta t\;f\left(u_{ij},\;t_{k}\right),\;\frac{d}{dt}u\left(x_{i},y,t\;\right)=f(u),$$ (22)

$$\overset{ij}{\underset{\text{2}}{R}}=\Delta t\;f(u_{ij}+\frac{\overset{ij}{\underset{\text{1}}{R}}}{\text{2}},\;t_{k}+\frac{\Delta t}{\text{2}})$$ (23)

$$\overset{ij}{\underset{\text{3}}{R}}=\Delta t\;f(u_{ij}+\frac{\overset{ij}{\underset{\text{2}}{R}}}{\text{2}},\;t_{k}+\frac{\Delta t}{\text{2}})$$ (24)

$$\overset{ij}{\underset{\text{4}}{R}}=\Delta t\;f(u_{ij}+\overset{ij}{\underset{\text{3}}{R}},\;t_{k}+\Delta t)$$ (25)

Where $t_{k}$ is primary time value expressed as, $\Delta t=t_{k+\text{1}}-t_{k},\;k=\text{0},\text{1},\text{2},..$The given unstable (or stable) fold’s value of u will not be subject to further analysis until it reaches its maximum or minimum.

#### 3.1.3. Stage III: Strong stability preserving Runge–Kutta method (SSP-RK54).

SSP scheme based on forward Euler time discretization, under adjusted time-step constraints. Also, SSP-RK54 technique up to five stages and order four preserves the strong stability properties, low storage implementation and less accumulation errors [46–52]. The details of SSP-RK54 method are offered as:


$$u^{\left(\text{1}\right)}=u^{n}+\lambda_{\text{1}}\Delta t\;f\left(u^{n}\right),\;\frac{d}{dt}u\left(x_{i},\;y_{j},t\right)=f(u)$$ (26)

$$u^{\left(\text{2}\right)}={\lambda_{\text{2}}u}^{n}+{\lambda_{\text{3}}u}^{\left(\text{1}\right)}+\lambda_{\text{4}}\Delta t\;f\left(u^{\left(\text{1}\right)}\right),$$ (27)

$$u^{\left(\text{3}\right)}={\lambda_{\text{5}}u}^{n}+{\lambda_{\text{6}}u}^{\left(\text{2}\right)}+\lambda_{\text{7}}\Delta t\;f\left(u^{\left(\text{2}\right)}\right),$$ (28)

$$u^{\left(\text{4}\right)}={\lambda_{\text{8}}u}^{n}+{\lambda_{\text{9}}u}^{\left(\text{3}\right)}+\lambda_{\text{1}\text{0}}\Delta t\;f\left(u^{\left(\text{3}\right)}\right),$$ (29)

$$u^{\left(n+\text{1}\right)}={\lambda_{\text{1}\text{1}}u}^{\left(\text{2}\right)}+{\lambda_{\text{1}\text{2}}u}^{\left(\text{3}\right)}+\lambda_{\text{1}\text{3}}\Delta t\;f\left(u^{\left(\text{3}\right)}\right)+{\lambda_{\text{1}\text{4}}u}^{\left(\text{4}\right)}+\lambda_{\text{1}\text{5}}\Delta t\;f\left(u^{\left(\text{4}\right)}\right)$$ (30)

Where:


$$\lambda_{\text{1}}=\text{0}.\text{391752226571890},\;\lambda_{\text{2}}=\text{0}.\text{444370493651235},\;\lambda_{\text{3}}=\text{0}.\text{555629506348765},$$

$$\lambda_{\text{4}}=\text{0}.\text{3}\text{6}\text{8}\text{4}\text{1}\text{0}\text{5}\text{9}\text{3}\text{0}\text{5}\text{0}\text{3}\text{7}\text{1},\;\lambda_{\text{5}}=\text{0}.\text{6}\text{2}\text{0}\text{1}\text{0}\text{1}\text{8}\text{5}\text{1}\text{4}\text{8}\text{8}\text{4}\text{0}\text{3},\;\lambda_{\text{6}}=\text{0}.\text{3}\text{7}\text{9}\text{8}\text{9}\text{8}\text{1}\text{4}\text{8}\text{5}\text{1}\text{1}\text{5}\text{9}\text{7},$$

$$\lambda_{\text{7}}=\text{0}.\text{2}\text{5}\text{1}\text{8}\text{9}\text{1}\text{7}\text{7}\text{4}\text{2}\text{7}\text{1}\text{6}\text{9}\text{4},\;\lambda_{\text{8}}=\text{0}.\text{1}\text{7}\text{8}\text{0}\text{7}\text{9}\text{9}\text{5}\text{4}\text{3}\text{9}\text{3}\text{1}\text{3}\text{2},\;\lambda_{\text{9}}=\text{0}.\text{8}\text{2}\text{1}\text{9}\text{2}\text{0}\text{0}\text{4}\text{5}\text{6}\text{0}\text{6}\text{8}\text{6}\text{8},$$

$$\lambda_{\text{1}\text{0}}=\text{0}.\text{5}\text{4}\text{4}\text{9}\text{7}\text{4}\text{7}\text{5}\text{0}\text{2}\text{2}\text{8}\text{5}\text{2}\text{1},\;\lambda_{\text{1}\text{1}}=\text{0}.\text{5}\text{1}\text{7}\text{2}\text{3}\text{1}\text{6}\text{7}\text{1}\text{9}\text{7}\text{0}\text{5}\text{8}\text{5},\;\lambda_{\text{1}\text{2}}=\text{0}.\text{0}\text{9}\text{6}\text{0}\text{5}\text{9}\text{7}\text{1}\text{0}\text{5}\text{2}\text{6}\text{1}\text{4}\text{7},$$

$$\lambda_{\text{1}\text{3}}=\text{0}.\text{0}\text{6}\text{3}\text{6}\text{9}\text{2}\text{4}\text{6}\text{8}\text{6}\text{6}\text{6}\text{2}\text{9}\text{0},\;\lambda_{\text{1}\text{4}}=\text{0}.\text{3}\text{8}\text{6}\text{7}\text{0}\text{8}\text{6}\text{1}\text{7}\text{5}\text{0}\text{3}\text{2}\text{6}\text{9},\;\lambda_{\text{1}\text{5}}=\text{0}.\text{2}\text{2}\text{6}\text{0}\text{0}\text{7}\text{4}\text{8}\text{3}\text{2}\text{3}\text{6}\text{9}\text{0}\text{6}$$

The values of a will give this method more accuracy. Consequently, this method will likely become increasingly popular as the desire for higher order methods emerges.

## 4. Innovative descriptive convection-diffusion behavior results

Here we introduce the main objectives for this research, novel numerical methods are presented which applied for solving CDE in (2 + 1) -dimensional. These mechanisms are DSC relies on ‘DLK’, ‘RSK’ and ‘RDK’ [53–62] with RK4 and SSP-RK54. DSC transforms governing equations into ordinary differential equations which solved by RK4 and SSP-RK54 schemes, which tackled via code in MATLAB that intended to obtain the arithmetic and numerical solutions within specific configuration ***(Intel(R)core (TM) i5-5200U CPU@2.20GHz).*** The convergence, accuracy, stability and efficiency of these mechanisms are based on the comparison between the obtained results and optimal ones. Statistical analysis namely Absolute errors, L_2_, L∞ errors and rate of convergence (ROC) are measured. The four discrete error norms defined as [62–64]:


$$Absolute\;Error\;(\varepsilon )=\left|f_{numerical}\left(x_{i},y_{j},t_{l}\right)-f_{exact}(x_{i},y_{j},t_{l})\right|$$ (31)

$$L_{\text{2}}\;Error=\;\sqrt{\Delta x\;\sum_{i,\;j=\text{1}}^{M_{x,\;}M_{y\;}}{\left(f_{numerical}\left(x_{i},y_{j},t_{l}\right)-f_{exact}(x_{i},y_{j},t_{l})\right)}^{\text{2}}}$$ (32)

$$L_{\infty }\;Error=\underset{\text{1}<i<M_{x,},\;\text{1}<j<M_{y}}{\text{max}}\left|f_{numerical}\left(x_{i},y_{j},t_{l}\right)-f_{exact}(x_{i},y_{j},t_{l})\right|$$ (33)

$$ROC=\frac{\text{log}\left(L_{\infty }(M_{\text{1}})\right)/L_{\infty }(M_{\text{2}}))}{\text{log}(M_{\text{1}}/M_{\text{2}})},$$ (34)

Before showing the numerical results, the function Ψ(x,  y) of primary value for two-dimensional CDEs is:


$$u(x,\;y,\;\text{0})=\Psi (x,\;y)=\text{exp}[\frac{-{\left(x-x_{\text{0}}\right)}^{\text{2}}}{\alpha_{x}}-\frac{{\left(y-y_{\text{0}}\right)}^{\text{2}}}{\alpha_{y}}]$$ (35)

Also, the value of constant Wi for 1 ≤ *i* ≤ 4 calculated by the best fit results as follows:


$$u(x,\;y,\;t)=\;\frac{\text{1}}{\text{1}+\text{4}t}\text{exp}[\frac{-{\left(x-x_{\text{0}}-\beta_{x}t\right)}^{\text{2}}}{\alpha_{x}(\text{1}+\text{4}t)}-\frac{{\left(y-y_{\text{0}}-\beta_{y}t\right)}^{\text{2}}}{\alpha_{y}(\text{1}+\text{4}t)}]$$ (36)

### 4.1. Validity of DSC-DQM results with RK4 and SSP-RK54

First, we present the values of parameters that control the accuracy, and efficiency of DSC based on three shape functions (DLK, RSK and RDK). These parameters are namely bandwidth, regularization parameter, the parameter T and grid size which are recorded in “Tables 1,2”. By comparison the obtained results with exact ones, it is noted that the best values of parameters for DSC are (2s + 1) = 5, γ = 5* g_x_,T > 20 and Mx X My = 5×5 as shown in “Table 1”. Also, the efficiency of these shapes is measured by calculating CPU time for each scheme with the best value of parameter (2s + 1), γ, T and M) where DLK = 4.714347 Sec, RSK = 4.659336 Sec and RDK = 4.598817Sec.

**Table 1:** Obtained outcomes of DSC relies on suggested method with diverse bandwidth (2S+1), regulation parameter γ, grid size *α*_*x*_= *α*_*y*_ = *β*_*x*_ = *β*_*y*_ = 1, *x*_*0*_ = *y*_*0*_ =0.5, *t* = 0.0006, *x* = *y* = 0.5.

Grid size	bandwidth	DSC-DLK		DSC-RSK				DSC-RDK			
	2S+1			γ =1* g_x_	γ =2* g_x_	γ =5* g_x_	γ =8*g_x_	γ =1* g_x_	γ =2*g_x_	γ =5* g_x_	γ =8*g_x_
5×5	3	1.0065		0.9964	1.0070	1.0080	1.0029	0.9975	1.0025	1.0025	1.0025
	5	1.0048		1.0006	1.0097	0.9976	0.9976	0.9976	0.9976	0.9976	0.9976
	7	1.0048		1.0007	0.9984	0.9977	0.9977	0.9976	0.9976	0.9976	0.9976
7×7	3	1.0074		1.0007	1.0002	1.0002	1.0006	0.9966	1.0003	1.0001	1.0001
	5	1.0043		1.0078	1.0008	0.9934	0.9932	0.9975	0.9978	0.9977	0.9977
	7	1.0048		1.0024	1.0016	1.0005	1.0005	0.9975	1.0002	0.9977	0.9977
9×9	3	1.0079		0.9976	1.0009	1.0008	1.0005	0.9994	1.0001	0.9981	0.9980
	5	1.0038		1.0006	1.0002	0.9977	0.9958	0.9962	0.9964	0.9975	0.9975
	7	1.0050		0.9979	1.0032	1.0090	1.0065	0.9972	0.9974	0.9975	0.9975
11×11	3	1.0084		0.9930	1.0631	1.0930	1.0972	0.9991	0.9981	0.9880	0.9880
	5	1.0034		1.0008	1.0023	0.9891	0.9826	0.9919	0.9923	0.9959	0.9958
	7	1.0057		1.0008	1.0009	1.0001	1.0003	1.0001	1.0001	0.9979	0.9978
Optimal [70]	0.9976										
CPU time	4.714347 Sec		4.659336 Sec					4.598817 Sec			

**Table 2:** Variation of the results by RDK with different parameters (T), RSK methods using SSP-RK54 scheme and optimal ones in [0*,*1]^2^ with various grid sizes *Mx X My* with *Mx* = *My* $\alpha_{x}=\alpha_{y\;}=\beta_{x}=\beta_{y}=1,x_{0}=y_{0}=0,t=0.0014,\;\left(2S+1\right)=5,\;\gamma =\left(5^{*}g_{x}\right),\;x=y=0.5$.

Grid points *Mx*	T							DSC-RSK
	1	2	5	10	20	50	100	
**5**	0.9202	0.9819	0.9972	0.9919	0.9881	0.9941	0.9941	0.9941
**7**	0.9511	0.9862	0.9885	0.9898	0.9900	0.9900	0.9900	0.9900
**9**	0.9455	0.9858	0.9877	0.9891	0.9931	0.9931	0.9931	0.9931
**11**	0.9237	0.9762	0.9707	0.9824	0.9924	0.9924	0.9924	0.9924
**13**	0.8690	0.9754	0.9718	0.9843	0.9946	0.9946	0.9946	0.9946
**15**	0.8373	0.9351	0.9185	0.9815	0.9919	0.9919	0.9919	0.9919
Optimal [77]			0.9945					

Also, “Table 2” introduces the accuracy of the obtained results via comparison of the results by RDK-SSP-RK54, RSK-SSP-RK54 methods and the optimal solution at different values of parameter T. Further, “Table 2” shows that the numerical results by RDK-SSP-RK54 technique are convergent, stable and accurate at T ≥ 20.

Secondly, “Tables 3–5” show stability due to convergence of DSCDQM. “Table 3” explains the efficiency and accuracy of DSC-DQM relies on three combined kernel types with RK4 and SSP-RK54 by calculating maximum absolute error (L_∞_) and CPU time. Also, “Table 3” shows the high accuracy of DSC-RSK and DSC-RDK results at diverse sizes from (4×4) to (30×30) with L_∞_ ≤ 10^−5^, while DLK-DSC presents precise results with L_∞_ ≤ 10^−4^ at size ≤ (10×10). As well as, SSP-RK54 scheme is more accurate than RK4. The experiment done via simulator reveals that CPU time for RK4 is less than SSP-RK54.

**Table 3:** *L∞* error norms in [0*,* 1]^2^ with and different grid points *Mx × My* with *Mx* = *My. α*_*x*_ *=0.1, α*_*y*_ = 0.01, *β*_*x*_ = *β*_*y*_ = 0.5, *x*_*0*_ = *y*_*0*_ =0.5, *t* = 0.0025,(2S+1) = 5, *γ* = (5^*^g_x_).

Grid points, M_x_	RK4			SSP-RK54		
	DSC-DLK	DSC-RSK	DSC-RDK, T = 20	DSC-DLK	DSC-RSK	DSC-RDK,T = 20
4	1.2073e-7	2.2114e-8	2.0413e-8	1.2073e-7	2.2114e-8	2.0413e-8
5	5.5557e-7	4.7749e-7	2.6038e-7	5.5557e-7	3.1279e-7	2.6038e-7
7	1.4981e-5	5.5497e-6	4.5820e-6	1.4981e-5	5.5497e-6	2.4705e-6
10	3.3999e-4	8.2207e-5	2.6351e-5	3.4180e-4	4.8956e-5	1.6616e-5
15	0.4175	2.6471e-4	2.1966e-4	0.4309	2.6472e-4	7.4949e-5
20	4.2959e4	5.2471e-4	4.2834e-4	2.0305e5	5.2503e-4	6.4751e-5
30	2.0310e16	1.1296e-4	3.3323e-5	8.2691e17	1.3738e-4	1.9693e-5
CPU DLK -RK4 at M_x_ = 4				0.078089 sec		
CPU RSK- RK4 at M_x_ = 4				0.082537 sec		
CPU RDK- RK4 at M_x_ = 4				0.076960 sec		
CPU DLK -SSP-RK54 at M_x_ = 4				0.119638 sec		
CPU RSK – SSP-RK54 at M_x_ = 4				0.103962 sec		
CPU RDK – SSP-RK54 at M_x_ = 4				0.106285 sec		

“Table 4” presents a comparative analysis of various proposed mechanisms and their corresponding grid sizes. This table appears that RDK with SSP-RK54 scheme is slightly more accurate than RSK with L_2_ error ≤ 10^−6^. As well as, DLK achieves accurate results with L2 ≤ 10^−5^ at plate size ≤ (10×10).

**Table 4:** *L2* error norms in [0*,* 1]^2^ with and different grid points *Mx × My* with *Mx* = *My. α*_*x*_ *=0.1, α*_*y*_ = 0.01, *β*_*x*_ = *β*_*y*_ = 0.5, *x*_*0*_ = *y*_*0*_ =0.5, *t* = 0.0025,(2S+1) = 5, *γ* = (5^*^g_x_).

Grid points M_x_	RK4			SSP-RK54		
	DSC-DLK	DSC-RSK	DSC-RDK, T = 20	DSC-DLK	DSC-RSK	DSC-RDK, T = 20
**4**	4.0243e-8	7.3712e-9	6.8044e-9	4.0243e-8	7.3712e-9	6.8044e-9
**5**	1.3890e-7	1.1938e-7	6.5095e-8	1.3890e-7	7.8199e-8	6.5095e-8
**7**	2.4973e-6	9.2507e-7	7.6376e-7	2.4973e-6	9.2507e-7	4.1180e-7
**10**	3.7837e-5	9.1459e-6	2.9347e-5	3.8039e-5	5.4473e-6	1.8489e-6
**15**	0.0300	1.9099e-5	1.5847e-5	0.0310	1.9100e-5	5.4071e-6
**20**	2.2734e3	2.8385e-5	2.3166e-5	1.1436e4	2.8402e-5	3.5017e-6
**30**	7.1290e14	4.3904e-6	1.4880e-6	3.8392e16	5.2466e-6	8.9261e-7

In addition, “Table 5” constructs convergent rate (ROC) at different grid size (5 × 5 ≤ M_x_ XM_y_ ≤ 30 × 30) for presented methods. Consequently, it is found that the rate of convergence (ROC) is less than 5 at plate size> (10×10) for RSK-SSP-RK54 and is less than 1 at plate size> (10×10) for RDK-SSP-RK54.

**Table 5:** ROC in [0*,* 1]^2^ with and various grid sizes *Mx × My* with *Mx* = *My α*_*x*_ *=0.1, α*_*y*_ = 0.01, *β*_*x*_ = *β*_*y*_ = 0.5, *x*_*0*_ = *y*_*0*_ =0.5, *t* = 0.005,(2S+1) = 5, *γ* = (5^*^g_x_).

Grid points M_x_	RK4			SSP-RK54		
	DSC-DLK	DSC-RSK	DSC-RDK, T = 20	DSC-DLK	DSC-RSK	DSC-RDK, T = 20
**5**	6.84	12.75	11.42	6.84	11.57	11.42
**7**	8.91	7.3	8.45	8.91	8.45	6.65
**10**	8.87	7.58	4.90	9.12	6.07	5.27
**15**	17.55	2.86	5.10	17.62	4.20	0.503
**20**	40.04	2.36	2.32	45.33	2.34	0.531
**30**	66.30	3.80	6.29	71.53	3.40	2.92

“Tables 1–5” take the values $\alpha_{x}=\text{0}.\text{1},\;\alpha_{y}={\text{0}.\text{01},\;\beta }_{x}=\beta_{y}=\text{0}.\text{5},\;x_{\text{0}}={\text{y}}_{\text{0}},\;\text{0}.\text{5},\;t=\text{0}.\text{0}\text{0}\text{5}$ Third, we solve this problem at t = 0.0004, x = y = 0.5 and $\alpha_{y}=\beta_{x}=\beta_{y}=\text{1}$ with different the values $\alpha_{x}$ and$\beta_{x}$ to see the accuracy and efficiency by another angle. The calculated outcomes for u are reported in “Tables 6,7”, which confirm that obtained outcomes by ‘RSK-SSP-RK54’ and ‘RDK-SSP-RK54’ patterns are well when seeking the optimal solution [78–80] at diverse values of 0.5 ≤ *α*_*x*_ ≤ 30 In “Table 6” and 0.5 ≤ *β*_*x*_ ≤ 10 In “Table 7”. From “Tables 6,7”, the results are inversely proportional with $\alpha_{x}$ and directly proportional with$\beta_{x}$. Also, from all “Tables 1–7” we found that the results by SSP-RK54 scheme is more slightly accurate and efficient than RK4.

**Table 6:** Comparisons between DSC kernels and optimal solution in [0*,* 1]^2^ with various $\alpha_{x}$ with *Mx* = *My* = 5. *α*_*y*_ = 1, *β*_*x*_ = *β*_*y*_ = 1, *x*_*0*_ = *y*_*0*_ =0, *t* = 0.0004,(2S+1) = 5, *γ* = (5^*^g_x_), x = y = 0.5.

$\alpha_{x}$	RK4			SSP-RK54			Optimal [78]
	DSC˗DLK	DSC˗RSK	DSC˗RDK T = 20	DSC˗DLK	DSC˗RSK	DSC˗RDK T = 20	
**0.5**	1.2872	1.2835	1.2835	1.2844	1.2835	1.2835	1.2838
**1**	1.0008	0.9996	0.9996	1.0001	0.9996	0.9996	0.9998
**1.5**	0.9203	0.9196	0.9196	0.9201	0.9196	0.9196	0.9199
**2**	0.8824	0.8820	0.8820	0.8825	0.8820	0.8820	0.8824
**5**	0.8186	0.8178	0.8178	0.8186	0.8178	0.8178	0.8186
**20**	0.7828	0.7886	0.7886	0.7880	0.7886	0.7886	0.7885
**30**	0.7766	0.7810	0.7810	0.7844	0.7810	0.7810	0.7852

**Table 7:** Comparisons between DSC kernels and optimal solution in [0, 1]^2^ with various $\beta_{x}$ with *Mx* = *My* = 5. *α*_*x*_ = *α*_*y*_, *β*_*y*_ = *1*, *x*_*0*_ = *y*_*0*_ =0, *t* = 0.004,(2S+1) = 5, *γ* = (5^*^g_x_), x = y = 0.5.

*β*_*x*_	**RK4**			**SSP-RK54**			Optimal [70]
	**DSC˗DLK**	**DSC˗RSK**	**DSC˗RDK** **T = 20**	**DSC˗DLK**	**DSC˗RSK**	**DSC˗RDK** **T = 20**	
**0.05**	1.0046	0.9802	0.9802	1.0046	0.9802	0.9802	0.9806
**0.5**	1.0063	0.9809	0.9809	1.0063	0.9809	0.9809	0.9823
**1**	1.0082	0.9817	0.9817	1.0082	0.9817	0.9817	0.9843
**1.5**	1.0101	0.9825	0.9825	1.0101	0.9825	0.9825	0.9862
**2**	1.0120	0.9834	0.9934	1.0120	0.9834	0.9934	0.9882
**5**	1.0231	0.9993	0.9993	1.0231	0.9993	0.9993	1.0003
10	1.0411	0.9922	0.9922	1.0411	0.9922	0.9922	1.0213

**Fig 3:**
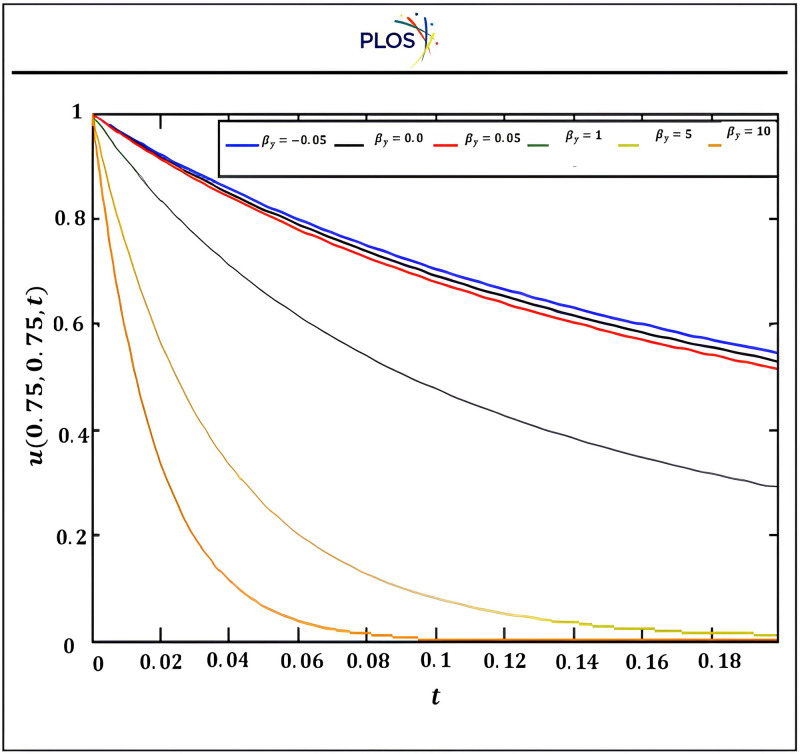
Numerical solution using RSK-SSPRK54 at$\alpha_{x}=\alpha_{y}=0.5,\;\beta_{x}=-0.08,\;x_{0}=y_{0}=0.5$ for several times.

**Fig 4:**
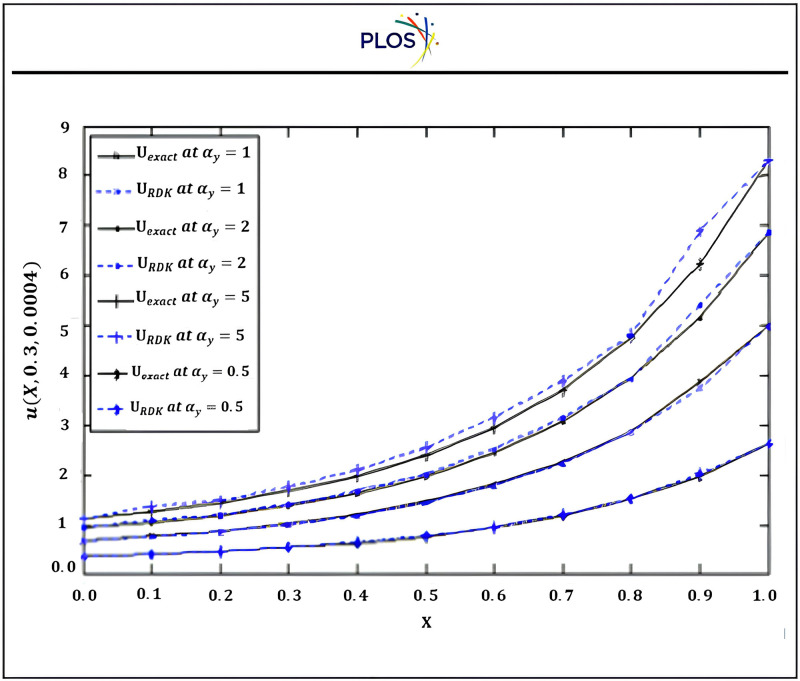
Numerical solution using RDK-SSP-RK54 at$\alpha_{x}=1,\;\beta_{x}=\beta_{y}=0.8,\;x_{0}=y_{0}=0.5$ for several locations.

Fourth, we introduce graphically the comparison between the results using three kernels with SSP-RK54 and optimal solution. “Fig 3” exhibits that the solution u is inversely proportional with time using RSK-SSPRK54. Also, “Fig 4” introduces the arithmetical solution using RDK-RK54 at several locations of x which is compared with optimal solution. In addition, this figure displays that the solution u is directly proportional with x and confirms that RDK-SSP-RK54 method is accurate at several values of $\alpha_{y}$.

**Fig 5:**
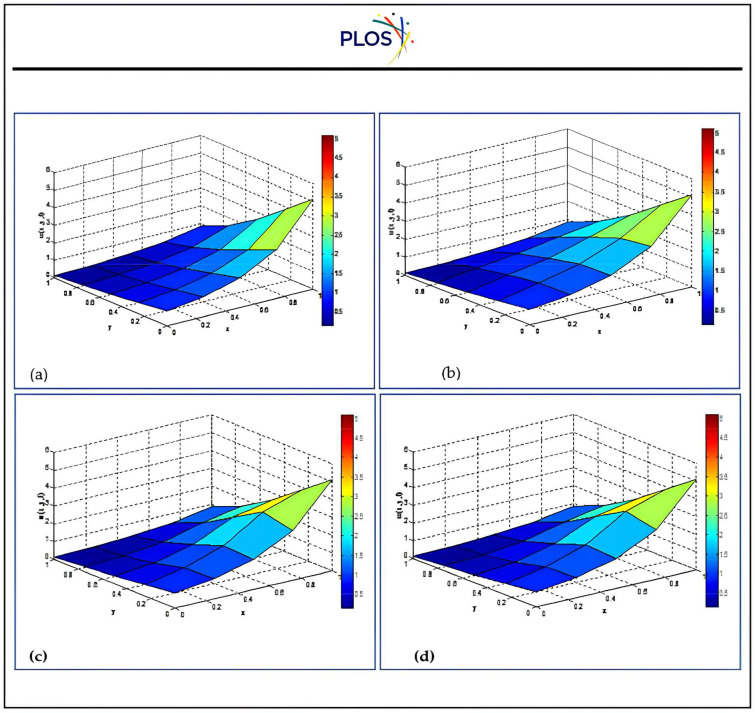
Physical behavior of proposed techniques compared to optimal solution at$\alpha_{x}=\alpha_{y}=1,\;\beta_{x}=\beta_{y}=0.8,\;x_{0}=y_{0}=0.5,\;t=0.04.\;$ a) Optimal b) DLK-SSPRK54 c) RSK-SSPRK54 d) RDK-SSPRK54.

**Fig 6:**
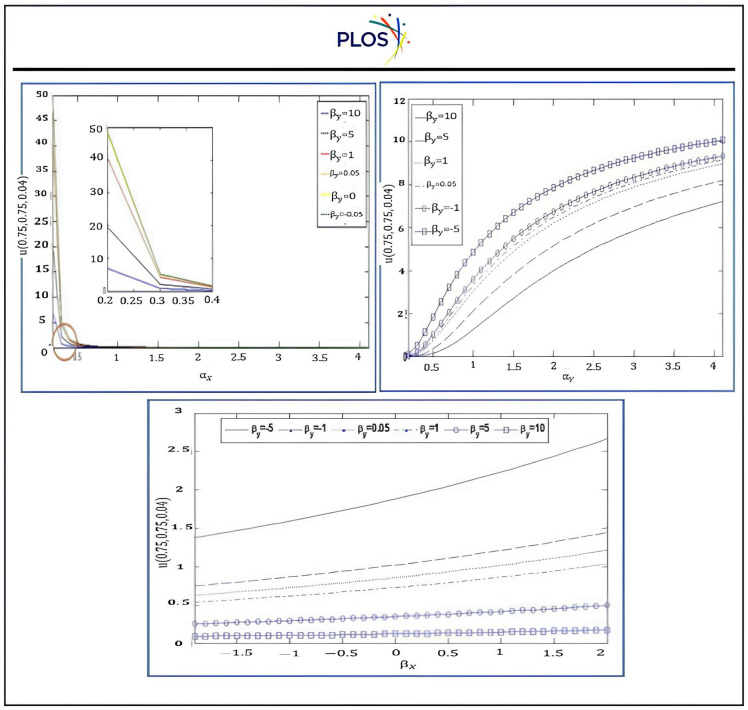
Contour plots of absolute errors of DLK-SSP-RK54 technique compared to optimal solution at*α*_*x*_ = *α*_*y*_= *β*_*x*_ = *β*_*y*_ = 1, *x*_*0*_ = *y*_*0*_ = 0.5, *t* = 0.6msec a) *Mx × My = 5 X 5* b) *Mx × My* *=* *9 X 9.*

Moreover, “Fig 5” shows that the solution behavior of all proposed schemes comparing to optimal solution at $\alpha_{x}=\alpha_{y}=\text{1},\;\beta_{x}=\beta_{y}=\text{0}.\text{8},\;x_{\text{0}}=y_{\text{0}}=\text{0}.\text{5},\;t=\text{0}.\text{0}\text{4}.$ Also, the absolute errors of DLK-SSP-RK54 solution behavior with $\alpha_{x}=\alpha_{y}=\beta_{x}=\beta_{y}=\text{1},\;x_{\text{0}}=y_{\text{0}}=\text{0}.\text{5},\;t=\text{0}.\text{0}\;$at different grid points Mx = My = 5, Mx = My = 9 are depicted in “Fig 6”.

**Fig 7:**
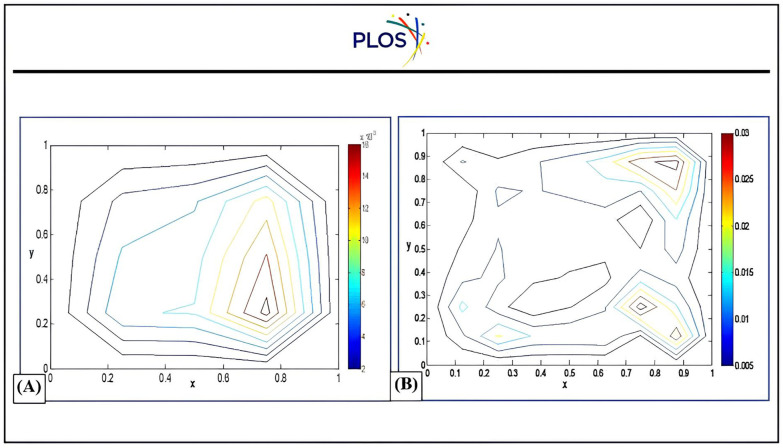
Influence of*α*_*x*_, *α*_*y*_, *β*_*x*_, *β*_*y*_ on the results using RDK-SSP-RK54 at *x*_*0*_ = *y*_*0*_ = 0.5. a) b) c).

From the “Tables 1–7” and “Fig 3–6” we observed that the results using DSCDQM-RDK with SSP-RK54 scheme is more accurate and efficient than other methods. So, we use this method to compute parametric studies. Finally, we present parametric study to investigate more effects of convective velocities, diffusion coefficients and time on results via RDK-SSP-RK54 scheme. “Fig 7” explains the influence of parameters $\alpha_{x},\;\alpha_{y},\;\beta_{x},\;\beta_{y}$ on numerical solution for RDK-SSP-RK54 at different values of $\alpha_{x},\;\alpha_{y},\;\beta_{x},\;\beta_{y}$ in intervals 0 ≤ x ≤ 1, 0 ≤ y ≤ 1.

**Fig 8:**
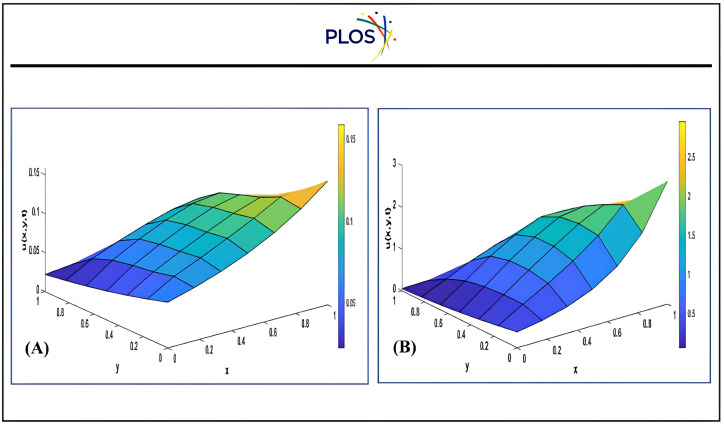
Distribution of 2- dimensional convection-diffusion equation using RD K-SS P-RK54 with*a*_*x*_ = *a*_*y*_ = 0.5, *β*_*x*_ = *β*_*y*_ = 0.8, *x*_*0*_ = *y*_*0*_ = 0.5 at different times a) Time = 0.4 b) Time = 4.

**Fig 9:**
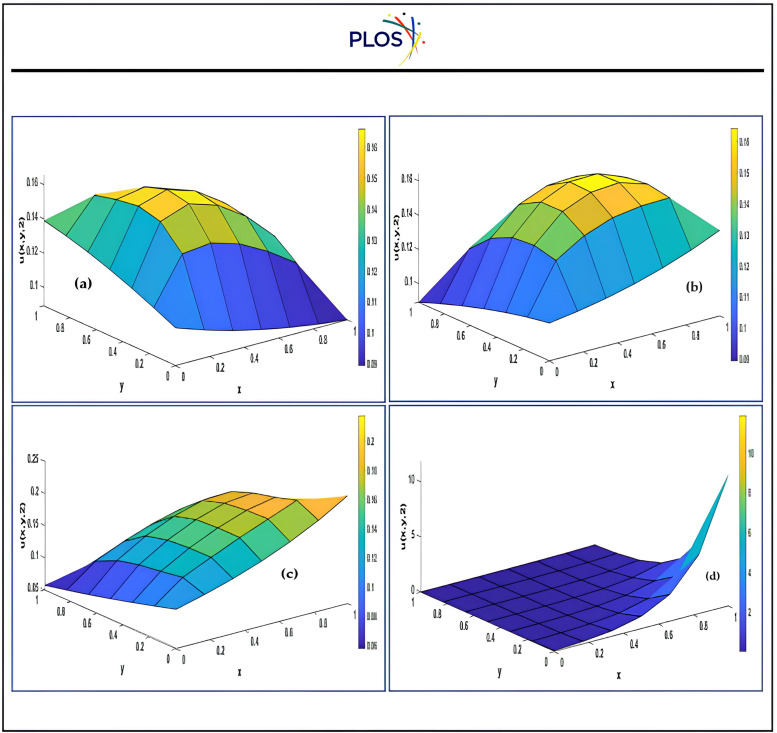
RDK-RK54 solution with*a*_*x*_ = *a*_*y*_ = 1, *x*_*0*_ = *y*_*0*_ = 0.5 at time = 2 a) *β*_*x*_ = *β*_*y*_ = -1 b) *β*_*x*_ = *β*_*y*_ = 0 c c) *β*_*x*_ = *β*_*y*_ = 1 d). *β*_*x*_ = *β*_*y*_ = 10.

“Fig 7” refers to the value of for u minimizes while rising $\alpha_{x},\;\beta_{y}$values and u increases with the values of $\alpha_{y},\;\beta_{x}$increase. The attitude of the numerical solution is tested at diverse timespan among t = 0.4 and t=4 seconds is depicted in “Fig 7” for *α*_*x*_ = *α*_*y*_ =0.5, *β*_*x*_ = *β*_*y*_ *=0.8*, *x*_*0*_ = *y*_*0*_ =0.5. The RDK-SSP-RK54 solution behavior with *α*_*x*_ = *α*_*y*_= 1, *x*_*0*_ = *y*_*0*_ = 0.5 at t = 2 is illustrated in “Fig 8 and 9” at different values for $\beta_{x},\beta_{y}$.

## 5. Phase II: eliminate defect causes during injection process

The elimination of defect reasons relies on the proactive prediction for the deviation in convection-diffusion behaviour out of the parameter that is set mathematically in the 1^st^ phase. There are four causes of defective plastic injected due to weakness in describing the convection-diffusion behaviour during blowing, injection, and drying processes. These causes occur due to mould surface temperature and lack of control of the output gate that are responsible for hot air expulsion, which is the motivation for seeking a high-accurate solution to program the control unit of the injection machine to resist causes of defects. There are four types of defects such as ***Flow lines*** $d_{f1}$**:** “These are the wavy patterns that appear on the narrow section of the molded parts due to the low mold temperature and the material temperature due to the lack of an accurate description of the convection diffusion behaviour”. ***Black Spots and burns*** $d_{f2}$*:* “Weld lines in plastic molded parts that cause the molten materials to converge on the surface of the assembly part, leading to surface burns due to the result of very low drying temperatures and very high molten materials”. ***Delamination*** $d_{f3}$*:* “The easy separation of thin layers on the surfaces of plastic molded parts is a sign of such delamination”. Delamination is an injection molding defect characterised by the peeling of the surface layer. It is a relatively serious type of defect that may reduce the strength of the molded component. This defect occurs due to excessive moisture. ***Warpage*** $d_{f4}$**:** “Deformation that occurs after the part is ejected from the mold due to weakness in the drying operation and not identified diffusion behaviour precisely”. Therefore, the proposed approach is divided into two stages. 1^st^ stage focuses on cooling the mould surface via coolant liquid controlling its feed rate via matching behavior of convection-diffusion under consideration, while the 2^nd^ stage focuses on sucking hot air from products to help in the drying process according to the behaviour of convection-diffusion distribution over the product surface [81].

“Table 8” shows Nine Steps pseudocode are utilized to estimate near-optimal values for crucial parameters, aiding in machine autonomous adaptation.

**Table 8:** The pseudocode of Mat-PYS controlling system.

$\vec{gap}=\text{zeros}({\text{L}}_{PYS},{\text{JRNS}}_{PYS})$ $weight=zeros\;(\text{1},\;{\text{L}}_{PYS})$ $\vec{direct}=\text{zeros}\left({\text{L}}_{PYS},{\text{JRNS}}_{PYS}\right)$ $\vec{\text{tar}}=zeros\;\left(\text{1},{\text{JRNS}}_{PYS}\right)$ $𝐟𝐨𝐫\;(\text{i}=\text{1}:{\text{L}}_{PYS})$ if f(i)≥f(tar) $weight(\text{1},i)=i^{(-\text{1})*\tau }$ $𝐟𝐨𝐫\;(\text{j}=\text{1}:{\text{JRNS}}_{PYS})$ $\vec{gap}(i,j)=\vec{\text{tar}}(\text{1},\text{j})-\vec{X}(\text{i},\text{j})$ $\vec{tar}(i,j)=\vec{\text{tar}}(\text{1},\text{j})-\vec{X}(\text{i},\text{j})*i^{(-\text{1})*\tau }$ **End** $𝐟𝐨𝐫\;(\text{d}=\text{1}:{\text{JRNS}}_{PYS})$ $\vec{direct}(i,d)=\text{sign}(\vec{\text{gap}}\;(\text{i},\text{d}))$ **end**	elseIff(i)≤f(tar) $if\;Rand\;(\;)\leq e^{\left[f(i)-f(tar)\right]}$ ${𝐟𝐨𝐫\;(\text{j}=\text{1}:\text{JRNS}}_{PYS})$ $\vec{gap}(i,j)=\vec{\text{tar}}(\text{1},\text{j})-\vec{X}(\text{i},\text{j})$ **end** $for\;(d=\text{1}:{\text{JRNS}}_{PYS})$ $\vec{direct}(i,d)=\text{sign}(\vec{\text{gap}}\;(\text{i},\text{d}))$ **end** **else** $for\;(d=\text{1}:{\text{JRNS}}_{PYS})$ $\vec{direct}(i,d)=\text{sign}(-\text{1}+(\text{1}+\text{1})*\text{Rand }(\;))$ **end** **end** **end** **end**

**Fig 10:**
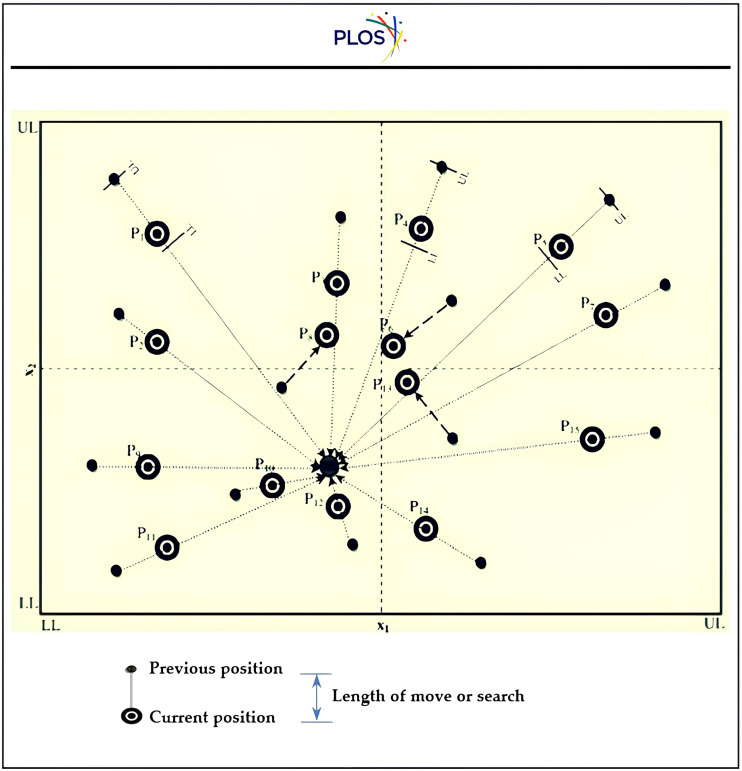
Moving on the meshes suggested via mathematical phase [77].

1-The first step involves setting up picking characteristics that align with the numerical solution obtained in the first phase with minimal error.
2-The process involves creating a predetermined number of first alternatives after preparing a parameter range and estimating the constraint.
3-Examine each individual’s respective state of fitness.
4-Examine the digital data library that instantaneously formed from the injection machine while working.
5-Check the poka-yoke list on the local dataset after crossing the cusp of the main JRNS mesh as illustrated in “Figs 5, 8, and 9” and discussed as illustrates in “Fig 10”. Where $L_{PYS}=\left\{\left({JRNS}_{pys},\varepsilon \right)\left|{JRNS}_{PYS}\right|,\;R^{N},\varepsilon ,R\right\}.$, ${JRNS}_{PYS}=[j_{\text{1}},j_{\text{2}},\ldots ,j_{i},\ldots j_{N}]$, R is the relationship order and N is the # of tested characteristics, which alternating among the user define factors in the Mat-PYS {${\alpha_{i},\beta }_{i},\;\gamma_{i},\;t$}. According to “Tables 1–7”.

Where, $L_{PYS}$ denote to number of poka-yoke significant factors and the dimensions of searching over${JRNS}_{PYS}.$

6-Search during JRNS meshes according to Eqn. (37)

$$f_{rs}=\delta_{\text{0}}+\sum_{i=\text{1}}^{N}\delta_{ii}n_{i}+\sum_{i=\text{1}}^{N}\delta_{ii}\overset{\text{2}}{\underset{i}{n}}+\sum_{i=\text{2}}^{N}\sum_{j=\text{1}}^{i-\text{1}}\delta_{ij}n_{i}n_{j}$$ (37)

7-Mat-PYS trained by $L_{PYS}$ to generate the conditional restrictions roughly
8-Find the reaction surfaces from “Tables 1–7” suitable to simultaneously picked data to decide suitable adaptive action.
9-Implement the pseudocode of Mat-PYS as shown in “Table 8”:

### 5.1. Stage I; Cooling mould surface

**Fig 11:**
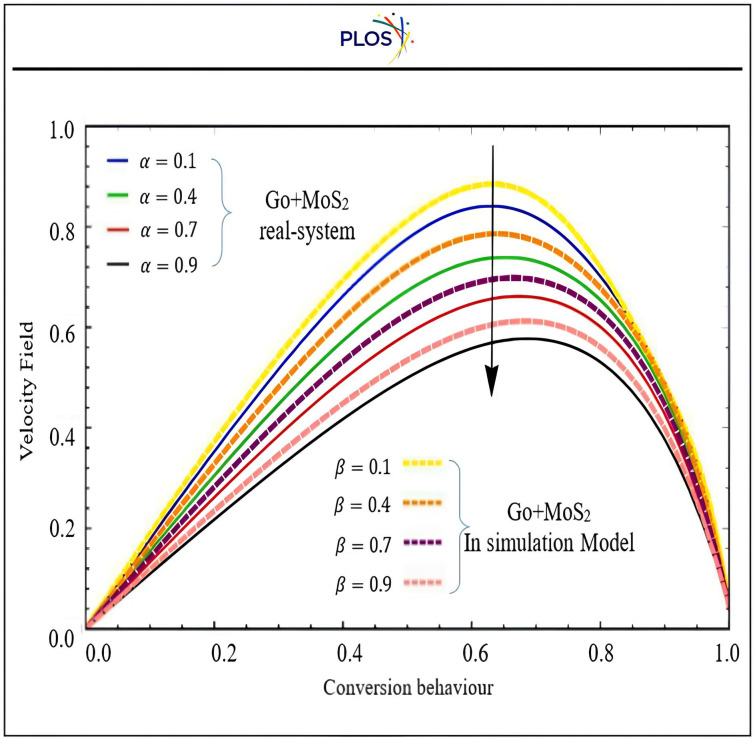
Impact of Fractional parametersα, β on concentration field at t = 0.4 at.ϕ=0.02

**Fig 12:**
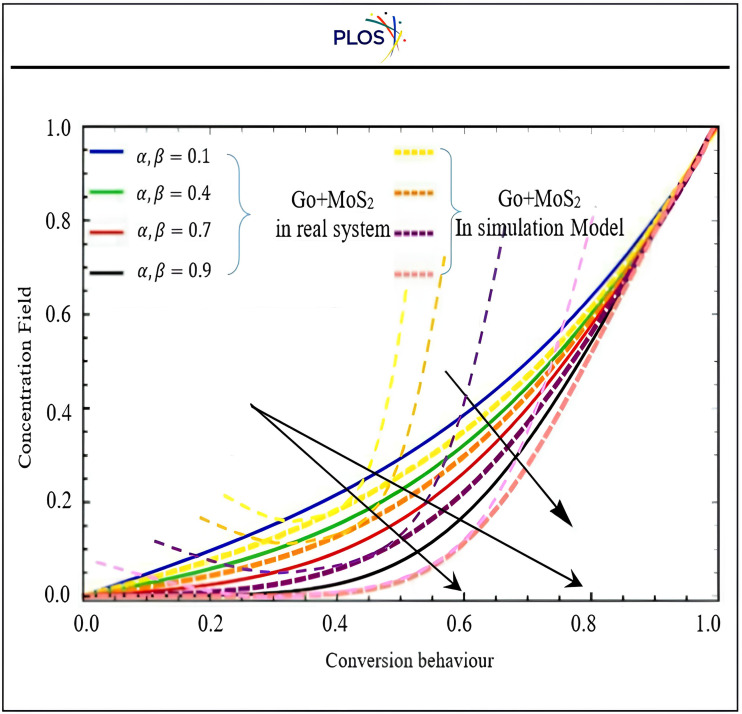
Influence of fractional on Fluid velocity (m/s) of conversion behaviour [${𝐰}_{\text{1}}(y,t)$] at Peclet number = 3.5, Heat and mass Grashof number = 2.6 and 4.5, when t = 0.4 and 0.02.


R. Jiwari et al., (2014) [82] and Abed, A. M. et al., (2024) [83] used PDEs with initial and boundary conditions as discussed in the first phase to solve the issue via the proposed transformation method, which is then used to analyse the results. The coolant liquid contains graphene oxide (GO) and molybdenum desulphated (MoS₂) nanoparticles because they have high thermal conductivity that are fed via the suggestion of convection-diffusion behaviour, which is basic to evaluate the thermal and hydrodynamic performance of hybrid nanofluids by calculating key performance parameters like Heat capacitance, Thermal Expansion Coefficient, and Thermal conductivity as expressed respectively in Eqns. (38),(39), and (40). This research test controls GO and MoS2 under the proposed mathematical model discussed in the 1st phase to measure coolant efficiency. The GO and MoS₂‘s nanoscale size enhances heat transmission due to improved interaction between nanoparticles and base fluids due to their large surface area-to-volume ratio. Building the industrial digital Mat-PY is not easy to control except get the function gained from 1st phase (**DSC-DQM-RSK** and **DSC-DQM-RDK**) and evaluate it via simulation to measure its reflection on products’ quality through 2nd phase and their two stages. The new hybridization accurately shows that the concentration field expands if a boost in the value of Pe slows down the momentum and temperature profiles. This forces us to reduce the number of fractional restrictions. The geometric structural results and the information available in Aman et al. (2018) [84] support the suggested (**DSC-DQM-RSK** and **DSC-DQM-RDK**). Additional support for the conclusions is provided by the interplay of the curves illustrations in Figs 9, 10, where α, β are fractional parameters for real system experiment and simulation tests during proposed digital Mat-PY model respectively. “Fig 11” displays the concentration fields of nanoparticles to match with diversity of convection-diffusion distribution, while “Fig 12” focuses on velocity required to hot air expulsion time. Both “Figs 11 and 12” compares real system of exact solution and proposed simulation model results that affect directly on 2^nd^ stage of this phase to tackle the suction output gate.

$${\left(\rho C_{p}\right)}_{nf}=\frac{{\left(\rho C_{p}\right)}_{hnf}}{\left(\text{1}-\phi_{\text{2}}\right)\left(\left(\text{1}-\phi_{\text{1}}\right)+\phi_{\text{1}}\frac{{\left(\rho C_{p}\right)}_{s\text{1}}}{{\left(\rho C_{p}\right)}_{f}}\right)+\phi_{\text{2}}{\left(\rho C_{p}\right)}_{s\text{2}}}$$ (38)

$${\left(\rho \beta \right)}_{f}=\frac{{\left(\rho \beta \right)}_{hnf}}{\left(\text{1}-\phi_{\text{2}}\right)\left(\left(\text{1}-\phi_{\text{1}}\right)+\phi_{\text{1}}\frac{(\rho \beta {)}_{s\text{1}}}{(\rho \beta {)}_{f\text{1}}}\right)+\phi_{\text{2}}(\rho \beta {)}_{s\text{2}}}$$ (39)

$$k_{f}=\frac{k_{bf}}{\left(\frac{k_{s\text{1}}+(n-\text{1})k_{f}-(n-\text{1})(k_{f}-k_{s\text{1}})\phi_{\text{1}}}{k_{s\text{1}}+(n-\text{1})k_{f}+(k_{f}-k_{s\text{1}})\phi_{\text{1}}}\right)}$$ (40)

### 5.2. Stage II; drying via sucking hot air

**Fig 13:**
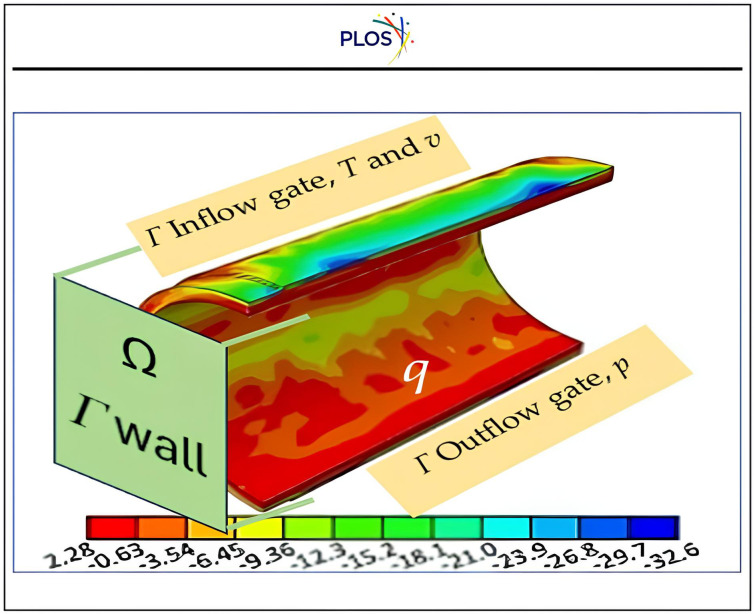
The topology optimization zone effect.

**Fig 14:**
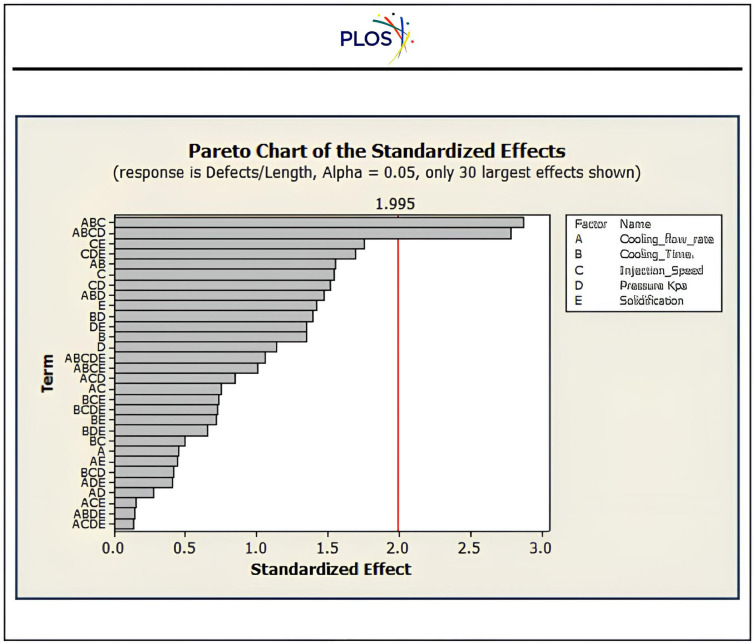
The significant variables effect the quality of product.

Innovative design techniques relying on convection-diffusion structural topology **Ω** optimisation that have been employed to create extremely efficient plastic pipe products via controlling the cooling systems in the injection machine to regulate the drying of plastic injection processes. This work presents a solution to optimise topology utilisation of convection-diffusion conformal cooling channels as discussed by *Samarskii, A. A. et al., (1993)* downstream toward *Jahan, S. et al., (2019) and Raza, O.V. Stadoleanu et al., (2024)* [85–87]. “Fig 13” illustrates the practical utilization of proposed solution that helps in control heat diffusion ***q***, at both gates of cooling system at Γinflow (absorbing hate) and Γoutflow of air to expel heat through Γwall boundary, and their properties such as velocity ***v***, cooling air temperature ***T***, and pressure ***p***. These parameters according to experiment shown as significance in quality of product as illustrated in “Fig 14”, which draw attention to controlling the flow rate of cooling air and their time, emphasises understanding significance of the behaviour of convection-diffusion to be compatible with drying process and resist defects.

Eqn. (40) describes the optimization in minimizing the effect of nodal heat flux ***q*** and nodal temperature ***T*** subject to proposed flow **DSC-DQM-(RSK or RDK)** that denoted by **D** that affect by velocity and friction force ***f*** that relies on the porosity $v_{e}\;$of each material during processed $\theta_{e}$ and care the whole volume fraction $V_{f}$ as expressed in Eqns. (41) and (42).


$$min\;Q^{c}=q^{T}T$$

$$\begin{array}{c} 𝐬.𝐭.[\begin{array}{cc} K & -D^{T}\\ -D & 0 \end{array}][\begin{array}{c} u\\ p \end{array}]=[\begin{array}{c} f\\ 0 \end{array}] \end{array}$$ (41)

$$\sum_{e=1}^{ne}v_{e}\theta_{e}\leq V_{f}$$ (42)

**Fig 15:**
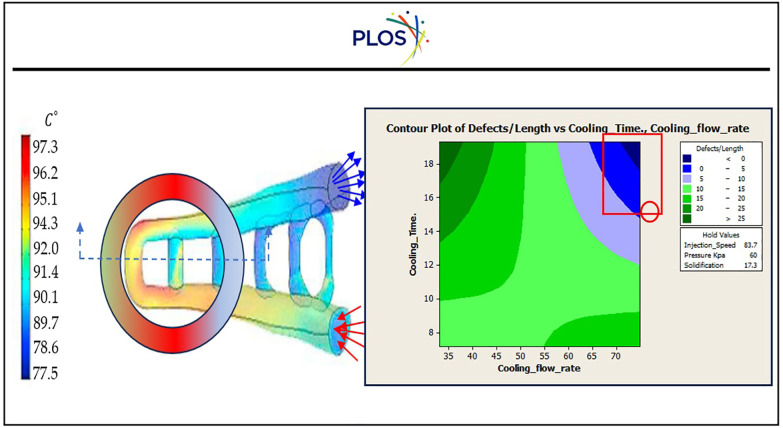
The suggested flow of convection-diffusion under control of DSC-DQM–(RSK or RDK).

The suggested design of cooling channels that face the Γwall boundary to absorb the heat with distribution matched with proposed mathematical solution suggested tested via built digital Mat-PY as illustrates in “Fig 15”.

In order to model and simulate the blowing and drying of plastic products, the density, rectangular shape, and size of the plastic raw material particles are determined to be compatible with the suitable convection and diffusion pattern as expressed in Eqn. (43). The experiment executes the particles that have density $kgm^{-\text{3}}$ and $V_{p}$express the volume of the pipe’s particles $m^{-\text{3}}$. The volume of the opened pores of the particles $V_{pores}$ and closed $V_{cp},\;m^{\text{3}}$ that hinder the drying process effect on the efficiency of utilizing the proposed mathematical model.


$$\rho_{p}=\frac{m_{p}}{V_{s}+V_{cp}+V_{pores}}=\frac{m_{p}}{V_{p}}$$ (43)

When examining drying processes, the moisture content of the plastic products during pipe blowing (i.e., before drying) is the primary consideration. The drying is absorbing the moisture until the sample weight becomes constant beneficiaries of the convective velocities and heat diffusion model. Therefore, dry basis expressed as in Eqn. (44), where $w_{b}$ and $w_{a}$ are the weight of product before and after drying (kg).


$$X=\text{1}\text{0}\text{0}\;(w_{b}-w_{a})/w_{a}$$ (44)

The moisture ratio MR relies on diffusivity $D_{eff}\;(m^{\text{2}}/s)$ follows Fick’s equation for spherical particles as expressed in Eqn. (45), where $X_{t}$ moisture at time t(sec.) to meet equilibrists (kg/kg) when initial moisture is $X_{\text{0}}$ and $F_{\text{0}}=D_{eff}\;(\frac{t}{\overset{\text{2}}{\underset{p}{th}}})$ represent Fourier number as cited in Serowik, M. et al., (2017) downstream to Feng, F., et al., (2025) [88,89], where $\overset{\text{2}}{\underset{p}{th}}$ is the thickness of blowing pipe.


$$MR=(X_{t}-X_{e})/(X_{\text{0}}-X_{e})=\frac{\text{6}}{\pi^{\text{2}}}\sum_{i=\text{1}}^{n}\frac{\text{1}}{n^{\text{2}}}\text{exp}\left(-n^{\text{2}}\pi^{\text{2}}F_{\text{0}}\right)$$ (45)

Over time, the equilibrium moisture content of the pipe being dried changes as a result of the drying air’s constant fluctuations in relative humidity based on the proposed model. “Table 9” demonstrates the experimentally determined values’ range between the proposed model **DSC-DQM-RSK** or **RDK** and experimental minimum blowing velocities $V_{ms}\;$with maximum drying pressure $\Delta P_{m}\;$to meet air inlet to compatible diffusion.

**Table 9:** The position of air inlet to $\;V_{ms}\;(\frac{m}{s})$ and $\Delta P_{m}\;(\frac{N}{m^{\text{2}}})$.

${𝐇}_{ms}$ (m) at angle slope30∘	Experimental measurements		DSC-DQM-RSK		DSC-DQM-RDK	
	${𝐕}_{ms}\;(\frac{m}{s})$	$\Delta P_{m}\;(\frac{N}{m^{\text{2}}})$	${𝐕}_{ms}\;(\frac{m}{s})$	$\Delta P_{m}\;(\frac{N}{m^{\text{2}}})$	${𝐕}_{ms}\;(\frac{m}{s})$	$\Delta P_{m}\;(\frac{N}{m^{\text{2}}})$
0.09	0.093	347.87	0.112	354.38	0.121	341.38
0.18	0.134	635.41	0.198	588.76	0.189	592.76
0.26	0.181	1183.34	0.205	1044.12	0.193	1141.21
0.31	0.193	1273.11	0.214	1190.48	0.201	1243.51

The mathematical model affects directly product quality when accurately describing the convection-diffusion equation via the **DSC-DQM-RSK, or RDK** model and helping in programming the machine to be efficient and increase their working uptime. At each stage of the production process, the availability (i.e., working time) Eqn. (46) that approximate to 98%, performance (P) Eqn. (47) that approximate 93.044%, and quality (i.e., avoid defect) Eqn. (48) that approximate to **97.5%** are multiplied to get the OEE score for plastic injection and tracks near to 88.9%. ${TL}_{ST}$: Production stoppages losses due to lack of control during dry step in the first and second stage of operation.


$$Availability\;=\frac{Ratio\;of\;Operating\;Time}{Planned\;working\;time}=\frac{T_{O}}{T_{P}}=\frac{T_{P}-{TL}_{ST}}{T_{P}}$$ (46)

$$Performance\;=\frac{Planned\;cycle\;time}{Average\;cycle\;time}=\frac{{CT}_{P}}{\overset{―}{{CT}_{O}}}=\frac{CT_{P}}{\left(\frac{T_{O}}{P_{O}}\right)}$$ (47)

$$Quality\;=\frac{\#\;of\;good\;product}{\#\;of\;total\;outputs}=\frac{P_{Q}}{P_{O}}=\frac{P_{O}-{PL}_{Q}}{P_{O}}$$ (48)

We used statistics from hypothesis testing to look at the production data to see how good the products were. “Table 10” shows the defective frequency occurrence in the batch represented by 1850 units of produced pipes due to lack of drying system control due to lack of accuracy in previous convection-diffusion function and after adopting the proposed new hybridisation titled by **DSC-DQM-RSK** and **DSC-DQM-RDK**. The total defective occurrences in the sample are 184 and 6 respectively for defective before and after implementing the controller underlying digital Mat-PY simulation model when adopting the proposed structural of mathematical functions.

**Table 10:** The defect per million opportunity occurrences for most defective types $d_{fi\;\forall \;i=1,2,3,4}$.

Total Occurrence in the sample (2850 random bottle)	${𝐝}_{f1}$	${𝐝}_{f2}$	${𝐝}_{f3}$	${𝐝}_{f4}$	
Before (as-is) situation without Poka Yoke control system	36	28	39	71	174
(Stage I), After: DSC-DQM-RSK +JRNS (Cooling mould surface)	2	2	1	1	6
(Stage II), After: DSC-DQM-RDK +JRNS (Sucking hot air)	1	0	0	1	2

The total defects per million can be computed by Eqn. (49.1) to determine the Sigma production level before adopting the proposed controller and Eqns. (49.2 and 49.3) to determine the sigma level after using proposed **DSC-DQM-RSK** and **DSC-DQM-RDK** that hybridize with JRNS in controlling the drying process during injection pipes through their two stages.


$$\frac{Total\;Occurance}{Number\;of\;defects\;\times sample\;size}\times {\text{1}\text{0}}^{\text{6}}=\frac{\text{1}\text{7}\text{4}}{\text{4}\times \text{2}\text{8}\text{5}\text{0}}\approx \text{1}\text{5}\text{2}\text{3}\text{6}\;unit/million$$ (49.1)

$$\frac{Total\;Occurance}{Number\;of\;defects\;\times sample\;size}\times {\text{1}\text{0}}^{\text{6}}=\frac{\text{6}}{\text{4}\times \text{2}\text{8}\text{5}\text{0}}\approx \text{5}\text{2}\text{6}\;unit/million$$ (49.2)

$$\frac{Total\;Occurance}{Number\;of\;defects\;\times sample\;size}\times {\text{1}\text{0}}^{\text{6}}=\frac{\text{2}}{\text{4}\times \text{2}\text{8}\text{5}\text{0}}\approx \text{1}\text{7}\text{5}\;unit/million$$ (49.3)

According to six sigma conversion-table [90] can determine the sigma level before to approximate 3.65 and 4.75 after controlling stage I (i.e., cooling mould surface) and meet sigma level 5.2 when controlling stage II (i.e., Sucking hot air) using proposed new hybridization model. The total productivity per day estimates by average 25267 unit/day in each shift.

“Table 10” shows that defective reduced by 97.5% of daily productivity were thrown away during production (drying steps) because the convection and diffusion during hot blowing and drying operations were not precisely controlled, which led to mistakes and losses (Eqn. (50)).


$$P_{Q}+{PL}_{ST}+{PL}_{SP}+{PL}_{Q}={P_{O}+PL}_{ST}+{PL}_{SP}=P_{O}+\text{0}+\left(\frac{T_{P}}{{CT}_{P}}-\frac{T_{P}}{\overset{―}{{CT}_{O}}}\right)$$ (50)

Prior to programming the plastic machine’s ROM unit to manage the drying stage in accordance with **DSC-DQM-RSK and RDK** behaviour, the OEE computation yielded better results than the OEE value that was determined the use of the recommended mathematical model as shown in “Table 11”. Eqns. (51) and (52) give the probability of scrap’s frequency and acceptance, respectively. In actuality, rework and repair are included in the waste creation rate. After then, the item’s quality data will be adjusted once more [79].

**Table 11:** OEE without and with using the proposed mathematical model and improve injection process.

Items	DSC-DQM-RSK +JRNS	DSC-DQM-RDK +JRNS	Items	DSC-DQM-RSK +JRNS	DSC-DQM- RDK +JRNS
	Both Cooling mold surface and sucking hot air stages				
$T_{P}$	322,560 mins.		${PL}_{Q}$	454.1 units	9.76 units
${TL}_{ST}$	11.67 mins.		${PL}_{ST}$	602 units	8 units
${CT}_{P}$	1.0834 mins.		${PL}_{SP}$	6 units	6 units
$\overset{―}{{CT}_{A}}$	1.16439 mins		Rework offline	36.1 units	7.9 units
$P_{A}$	22914 units/day	29129 units/day	$Q_{S}\%$	0.093%	0.00031%
$P_{Q}$	1367 units/day	1367 units/day	$Q_{A}\%$	0.882%	0.8354%
Unexplained loss	6.1 units.	zero	Misjudgment of inspection	2 units.	2 units.


$$Q_{S}=\text{2}\left(\text{1}-{\int \phi_{x}(x)d_{x}}_{|x-\alpha |\leq \frac{T}{\text{2}}}\right)\int_{\text{0}}^{\frac{T}{\text{2}}}\phi_{Z\text{1}}(Z)d_{z}$$ (51)

$$Q_{A}=\text{2}\left(\text{1}-{\int \phi_{x}(x)d_{x}}_{|x-\alpha |\leq \frac{T}{\text{2}}}\right)\int_{\text{0}}^{\frac{T}{\text{2}}}\phi_{Z\text{2}}(Z)d_{z}$$ (52)

**Fig 16:**
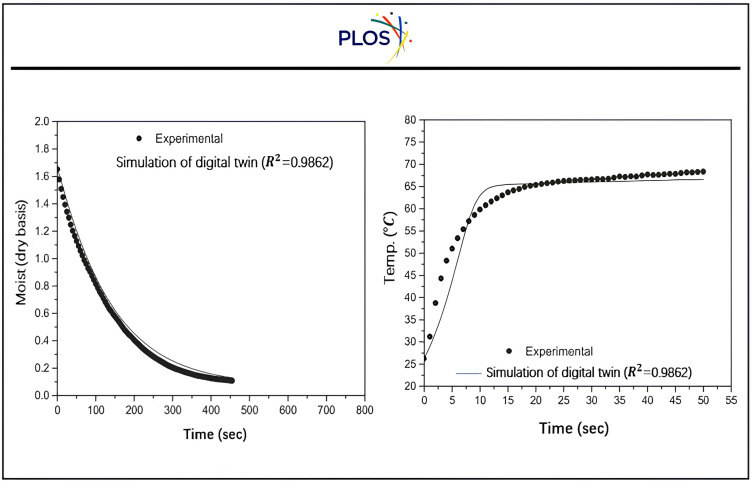
Experimental moist and Temperature data predicted by a suggested mathematical model as a function of time through digital twin simulation.

Based on empirical data regarding temperature, the mathematical model predicted the intake ambient temperature related to time. Through simulation and comparison with experimental data, the effectiveness of the mathematical model for estimating particle and pipe moist content was assessed. The simulation of the suggested model for the temperature and moist data during the drying process is illustrated in “Fig 16” as a mimic to Bash, A. A. K., & Khail, A. A. (2025) and L. D. Minh et al., (2023) [91,92].

**Fig 17:**
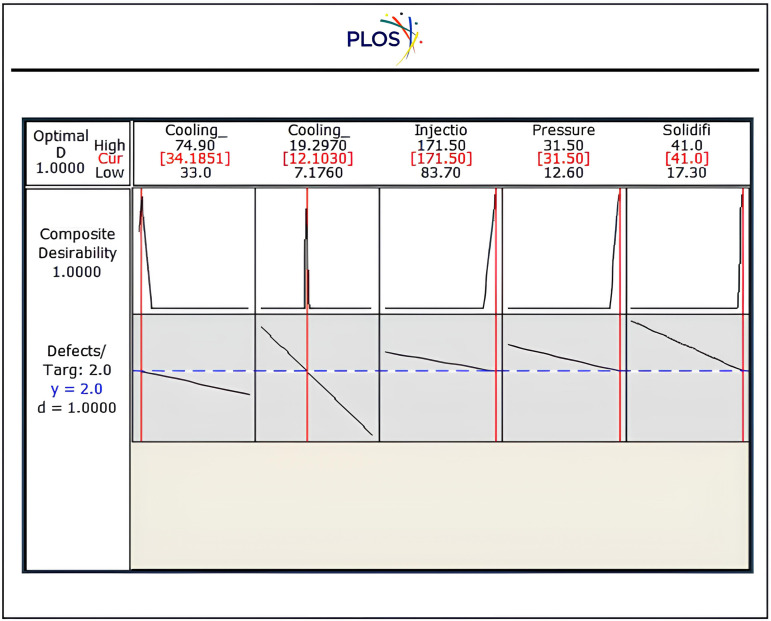
The Optimisation of the injection controller.

“Fig 17” illustrates Optimisation of the injection controller of the drying process to eliminate wrong products due to lack of description of convection-diffusion behaviour. When set the digital Mat-PY simulator with these tuning especially cooling time at 12.1030 sec, while pressure approximate at 31.5 Kpa find that sigma level of productivity approximate to 4.4 as shown in “Table 12”, which lists the measurements that were taken during the experimental testing for the inflow and outflow gates to control convection-diffusion behaviour and related OEE% that increased by 11.5%, which reflected positively on increased quality products [93,94].

**Table 12:** Comparison before and after programing digital Mat-PYS with proposed convection-diffusion mathematically behaviour.

Significant parameters	Before	After	Significant parameters	Before	After
Temp of the coolant inflow	97.3 $C^{\circ }$	81.76 $C^{\circ }$	The velocity of coolant transfer	6.471 L/min	6.471 L/min
Temp of the coolant outflow	77.5 $C^{\circ }$	85.00 $C^{\circ }$	Pressure in the coolant	8.53 kPa	9.36 kPa
Volume of injection compressed	4.35 Mpa	4.35 Mpa	Fixing power of clamping	1.152 MN	1.213 MN
Total takt time	43.12 Sec.	33.18 Sec.	Cooling period time	22 Sec.	12.1 Sec.
DPMO (Defect per Million Opportunity)	1242/$m^{\text{3}}$	102/$m^{\text{3}}$	Rework offline **DSC-DQM-RSK**	264 units	36.1 units
The sample 2850 units.	3.65	5.2	Rework offline **DSC-DQM-RDK**	85 units.	2 units

The proposed Mat-PYS validity was done via comparing the optimal prediction of deviation when working conditions of the injection process on the generated meshes, as illustrated in “Figs 5, 8, and 9”. As indicated in “Table 13”, the primary goal of hybridisation is to enhance machining performance by rapidly modifying the machine to avoid the emergence of defect chances. “Table 13” uses coloring to indicate the lowest values that indicate the best option. Select most ten functions that superior in published ref. [70] are tried, the faults before and after using published data of JRNS [70] and test similar effective mathematical and heuristic algorithm use ant colony that tuned and improved mathematically as discussed in [95] implementing the proposed Mat-PYS are indicated by the ($d_{B}$,$d_{A,\;JRNS}$, $d_{A,Mat-PYS},\;d_{AMat-ACO}$). Although the analysis showed that the Mat-PYS performed better in particular use zones.

**Table 13:** The comparison of fifteen mechanisms with the proposed digital Mat-PYS simulator [95].

	Comparing functions and methods	Range	(${𝐝}_{B}$,${𝐝}_{AJRNS}$,${𝐝}_{AMat-PYS,\;}$ ${𝐝}_{AMat-ACO}$)	D	f(S*) *of response time*	*Most Defect controlled*	*Results discovering time*	Mat-ACO	JRNS	Mat-PYS
1	Cozine Mixture Problem (CM), Uni Modal	[-1.2:1.2]	**(2850, 97, 26, 11)**	2,4	0.4	$d_{f3}$	**best**	**0.399**	**0.4**	**0.33**
							**Mean**	**0.398**	**0.38**	**0.33**
							**Std-Dev(S**_**td**_)	**7.050E** − 4	**1.412E-16**	**1.323E-17**
							**%**	**97**	**26**	**11**
2	Epistatic Michal−ewicz behavior (EM), Multi Modal	[0: Pi]	**(2850, 339, 54, 44)**	5	−9.66	$d_{f1}$,$d_{f3}$	**best**	**−9.527**	**−6.2643**	**−7.386**
							**Mean**	**−9.146**	**−6.317**	**−5.793**
							**Std-Dev(S**_**td**_)	**0.226**	**5.01**	**4.01**
							**%**	**399**	**54**	**44**
3	Exponential−behavior (EXP), Uni Modal	[−1:1]	**(2850, 103, 30, 7)**	10	1	$d_{f1}$, $d_{f2}$	**best**	**0.992**	**1.05**	**1.025**
							**Mean**	**0.985**	**1**	**0.9**
							**Std-Dev(S**_**td**_)	**0.0045**	**0**	**0**
							**%**	**103**	**30**	**7**
4	Neumaier 3−behavior (NF3),Multi Modal	[-D^2^:D^2^]	**(2850, 117, 67, 13)**	10	−210	$d_{f4}$, $d_{f1}$	**best**	**−209.910**	**−147.01**	**−134.41**
							**Mean**	**−208.398**	**−117.71**	**−109.746**
							**Std-Dev(S**_**td**_)	**1.96285**	**56.945**	**45.85355**
							**%**	**117**	**67**	**13**
5	Paviani’s−behavior Eq. (PP), Uni Modal	[2:10]	**(2850, 196, 78, 18)**	10	−45.78	$d_{f3}$, $d_{f2}$	**best**	**−45.76**	**−27.79**	**−23.79**
							**Mean**	**−45.74**	**−26.39**	**−21.39**
							**Std-Dev(S**_**td**_)	**0.014**	**4.058**	**3.058**
							**%**	**196**	**78**	**18**
6	Odd Square−behavior (OSP), Multi Modal	[−15:15]	**(2850, 177, 59, 29)**	10	−1.144	$d_{f3}$, $d_{f1}$	**best**	**−0.0049**	**−0.3154**	**−0.3753**
							**Mean**	**−0.0022**	**−0.2916**	**−0.2234**
							**Std-Dev(S**_**td**_)	**9.081E − 4**	**9.081E − 5**	**8.081E − 5**
							**best**	**177**	**59**	**29**
7	Ackley’s−behavior (ACK), Multi Modal	[−31:31]	**(2850, 11, 44, 3)**	10	0	$d_{f3}$, $d_{f2}$, $d_{f4}$,$d_{f1}$	**Mean**	**0.013**	**7.3E-15**	**8.3E-17**
							**Std-Dev(S**_**td**_)	**0.054**	**7.3E-15**	**8.8E-17**
							**%**	**0.054**	**0.014**	**0.011**
							**best**	**11**	**44**	**3**
8	Griewank behavior (GW), Multi Modal	[−700:700]	**(2850, 150, 49, 19)**	10	0	$d_{f3}$, $d_{f2}$, $d_{f4}$,$d_{f1}$	**Mean**	**0.325**	**0**	**0**
							**Std-Dev(S**_**td**_)	**0.765**	**0**	**0**
							**%**	**0.266**	**0**	**0**
							**best**	**150**	**49**	**19**
9	Rastrigin−behavior (RG), Multi Modal	[-5.12:5.12]	**(2850, 141, 18, 8)**	10	0	$d_{f3}$, $d_{f2}$, $d_{f1}$,$d_{f4}$	**Mean**	**0.039**	**0**	**0**
							**Std-Dev(S**_**td**_)	**0.159**	**0**	**0**
							**%**	**0.095**	**0**	**0**
							**best**	**141**	**18**	**8**
10	Four−peak function, Uni Modal	[−140:140]	**(2850, 69, 14,3)**	10	0	$d_{f3}$, $d_{f2}$, $d_{f4}$,$d_{f1}$	**Mean**	**0.09987**	**0**	**0**
							**Std-Dev(S**_**td**_)	**0.20668**	**0**	**0**
							**%**	**0.0904**	**0**	**0**
								**69**	**14**	**3**

## 6. Conclusions

The elimination of defect reasons relies on the proactive prediction for the deviation in convection-diffusion parameters behaviour that are set mathematically and supported by a machine learning system called JRNS to meet accuracy and speed in decision making of maintaining the injection process in high-quality conditions. The proposed Mat-PY simulator model programmed via MATLAB under configuration (Intel(R), Core^(TM)^ i5-5200U CPU@2.20GHz) presents a fast and accurate numerical algorithm to solve the formulated behavior of (2 + 1) dimensional convection-diffusion equation. This algorithm is DSC that integrates with three new shape functions called DLK, RSK, and RDK, which are responsible for transforming the governing equations into a system of ODEs in time. The primary system value is solved by two schemes: the first is RK4 and SSP-RK54. The motivation is to improve plastic injection machining to resist defects that lead to malfunction of machines and lose productivity represented by ≈2.5% of total productivity per month. Automated control of the machine to adapt the manufacturing process parameters of machine parts to resist most defects such as flow line, trapped air, delamination, and the Warpage. The proposed system consists of two sequential phases. The first is interested in formulating the mathematical behavior of convection-diffusion during dry process that required cooling mould surface and sucking hot air as discussed in the 2^nd^ phase. The accuracy, convergence, and stability of obtained results at mesh sizes (5×5) to (11x11), bandwidth (2S+1) = 5 are shown in the forms of tables and figures, which emphasize the success of 1^st^ phase of Mat-PYS, when elected DSC-DQM-RDK and DSC-DQM-RSK that distinguish with low error ≤ 1×10^-5^ when hybridized with JRNS (Jidoka recurrent network system). Numerical results using three schemes are compared with the optimal ones of exact solution [37–43,51–56,90–93]. The Mat-PYS is a reliable, stable, accurate and efficient control system at regularization parameter $\gamma =\text{5}*g_{x}$ and the parameter T=20 in response time ≤ 0.4 second. Understanding the behavior of convection propagation and decision-making speed via the Mat-PYS quickly for distributing drying gates (i.e., inflow and outflow air) and determining drying time and air velocity with appropriate pressure to raise the product quality. The superiority of the 1^st^ phase (Mathematical formulation) is discussed in Tables 1–7 and “Figs 3–9” via four statistical analyses applied such as Rate of convergence, absolute error, L_2_ and L_∞_ errors to reliability and stability of obtained results. Also compared the response of adaptive to maintain the production process in high-quality conditions are discussed in “Tables 11–13”” and “Fig 17” when compared with Mat-ACO and solo JRNS algorithms. The OEE rose to 88.9% according to Eqns. (45–47). Data is restricted to a certain sector: The results may not be fully relevant to other sectors with various production conditions because this study focused on the plastic injection molding business, particularly in drying phases related to convection and diffusion patterns. However, this does not preclude the mathematical model from being used for the same objective in other domains. Suggestion for future research: To evaluate the method’s wider application, future studies should try this model in other production sectors like semiconductors (drying electronic components over extended working times) or automobiles (drying the color coat). The accuracy of solution u increased when $x,\;y,\beta_{x}\;$and $\alpha_{x}$ are increased and has direct effect on convective velocities, diffusion coefficients, time at different locations on the outcomes. Also, this work illustrated the ability of this method to solve linear and non-linear issues emerging in the areas of science and engineering.

## 7. Future work

Data is restricted to a certain sector: The results may not be fully relevant to other sectors with various production conditions because this study focused on the plastic injection moulding business, particularly in drying phases related to convection and diffusion patterns. However, this does not preclude the mathematical model from being used for the same objective in other domains. Suggestion for future research: To evaluate the method’s wider application, future studies should try this model in other production sectors like semiconductors (drying electronic components over extended working times) or automobiles (drying the color coat). We point to improving the control system that tackle the significant working parameters via describing the diffusion-convection through its PDE and extracting its numerical solution for significant working parameters via transferring the PDE to ODE to simplify its programming with minimum errors.

### Glossary: List of abbreviation and symbols

**Table 14:** 

Γ	Computational domain	2S+1	Computational bandwidth.
u(x, y, t)	Heat or vorticity	γ	Regularization parameter
α_x_ > 0, α_y_ > 0	Diffusion coefficients	d	Computational parameter
β_x_ and β_y_.	Convective velocities	*gx*	Step size
$\overset{x}{\underset{ik}{\text{P}}}$	1^st^ weighting coefficients	RK4	Runge-Kutta 4^th^ order
$\overset{xx}{\underset{ik}{\text{P}}}$	2^nd^ weighting coefficients	SSP-RK54	Strong stability preserving Runge–Kutta
Φ(t−X)	Singular kernel	DLK	delta Lagrange kernel
DLK	Delta Lagrange Kernel	RDK	Regularized Dirichlet kernel
RSK	Regularized Shannon kernel	ROC	Rate of convergence
HPM	Homotopy perturbation method	DQM	Differential quadrature method
$V_{p}$	Volume of the pipe$m^{-\text{3}}$	$V_{cp}$	Closed pores particles$\;m^{\text{3}}$
$V_{pores}$	Opened pores particles$\;m^{\text{3}}$	$Q_{S}$	Scrap’s products frequency
$m_{p}$	Mass of particles	$Q_{A}$	Products acceptance
